# Long-term organic carbon preservation enhanced by iron and manganese

**DOI:** 10.1038/s41586-023-06325-9

**Published:** 2023-08-02

**Authors:** Oliver W. Moore, Lisa Curti, Clare Woulds, James A. Bradley, Peyman Babakhani, Benjamin J. W. Mills, William B. Homoky, Ke-Qing Xiao, Andrew W. Bray, Ben J. Fisher, Majid Kazemian, Burkhard Kaulich, Andrew W. Dale, Caroline L. Peacock

**Affiliations:** 1 School of Earth and Environment, University of Leeds, Leeds, UK; 2 School of Geography, University of Leeds, Leeds, UK; 3 School of Geography, Queen Mary University of London, London, UK; 4 Department of Geochemistry, GFZ, German Research Centre for Geosciences, Potsdam, Germany; 5 Research Center for Eco-Environmental Sciences, Chinese Academy of Sciences, Beijing, China; 6 School of GeoSciences, University of Edinburgh, Edinburgh, UK; 7 Diamond Light Source Ltd., Harwell Science and Innovation Campus, Didcot, UK; 8 GEOMAR Helmholtz Centre for Ocean Research Kiel, Kiel, Germany

**Keywords:** Carbon cycle, Geochemistry, Marine chemistry

## Abstract

The balance between degradation and preservation of sedimentary organic carbon (OC) is important for global carbon and oxygen cycles^1^. The relative importance of different mechanisms and environmental conditions contributing to marine sedimentary OC preservation, however, remains unclear^2–8^. Simple organic molecules can be geopolymerized into recalcitrant forms by means of the Maillard reaction^5^, although reaction kinetics at marine sedimentary temperatures are thought to be slow^9,10^. More recent work in terrestrial systems suggests that the reaction can be catalysed by manganese minerals^11–13^, but the potential for the promotion of geopolymerized OC formation at marine sedimentary temperatures is uncertain. Here we present incubation experiments and find that iron and manganese ions and minerals abiotically catalyse the Maillard reaction by up to two orders of magnitude at temperatures relevant to continental margins where most preservation occurs^4^. Furthermore, the chemical signature of the reaction products closely resembles dissolved and total OC found in continental margin sediments globally. With the aid of a pore-water model^14^, we estimate that iron- and manganese-catalysed transformation of simple organic molecules into complex macromolecules might generate on the order of approximately 4.1 Tg C yr^−1^ for preservation in marine sediments. In the context of perhaps only about 63 Tg C yr^−1^ variation in sedimentary organic preservation over the past 300 million years^6^, we propose that variable iron and manganese inputs to the ocean could exert a substantial but hitherto unexplored impact on global OC preservation over geological time.

## Abstract (web summary)

Catalysis of simple organic carbon molecules into complex macromolecules by Fe and Mn may play a fundamental role in organic carbon preservation, to a degree that could substantially affect the Earth’s carbon and oxygen cycles.

## Main

The preservation of organic carbon (OC) in marine sediments over geological time requires that OC escapes microbial remineralization that otherwise converts it into dissolved inorganic carbon and/or carbon dioxide^7^. This premise is central to all OC preservation mechanisms and requires that OC is either inherently stable or is made stable against microbial breakdown^7^. The latter pathway to preservation is most often associated with the interaction of OC with mineral matrices^4,8^, but other routes may also involve the transformation of OC from labile to recalcitrant forms^5^. The Maillard reaction^15^ is one such route as it can polymerize any reducing sugar and free amino acid into complex aromatics (more than 1,000 g mol^−1^) possessing N-substituted rings, carbonyl, carboxyl and amino functional groups^16^ (Supplementary Fig. 1). These aromatic polymers, which we define as geopolymerized substances (GPS), are too large to be directly ingested by microbes and are more difficult to hydrolyse outside their cells (if more than 1,000 g mol^−1^) because they have more complex structures and so may escape microbial remineralization^17^ and thus persist in the environment over long timescales.

For geopolymerization to make a notable contribution to OC preservation in marine sediments, Maillard reaction kinetics must compete with microbial uptake or remineralization of reducing sugars and amino acids^7^. Maillard reaction kinetics at marine sediment temperatures (around 10 °C)^14^, however, are thought to be extremely slow^9,10^. As a result, geopolymerization has been largely discounted as a mechanism for OC preservation and assumed to be of only minor importance for OC burial in marine sediments^7,9,18^. More recent work, however, shows that the Maillard reaction can be catalysed at soil temperatures (25–45 °C) by the Mn mineral birnessite^11^ and clays^12^, leading to increased production of humic substances, which resemble those found abundantly in the soil environment. Moreover, in marine and terrestrial systems, the cycling of OC is known to be tightly coupled to the cycling of dissolved Fe and Mn, and mineral Fe and Mn (oxyhydr)oxides^13,19,20^, suggesting that these reactive forms of Fe and Mn may complex with OC molecules, helping to protect these molecules from remineralization and to preserve them over hundreds to thousands of years^8,19^. Even though Fe and Mn could play an important role in the transformation and preservation of OC, the potential of Fe and Mn to catalyse the Maillard reaction and promote the formation of geopolymerized OC at marine sediment temperatures has never been determined.

We incubated common organic molecules with dissolved Fe and dissolved Mn under anoxic conditions, as well as mineral Fe (oxyhydr)oxide (ferrihydrite) and mineral Mn oxide (birnessite) under oxic conditions, to determine their catalytic effect on the Maillard reaction of a representative dissolved reducing sugar (glucose) and a representative dissolved free amino acid (glycine) at reaction temperatures (10 °C) applicable to continental margin sediments. We found that the products of our experiments are consistent with the chemical signature of dissolved OC and total OC present in continental margin sediments from a spatially and temporally diverse sample set. We also found that these reactive forms of Fe and Mn catalyse the Maillard reaction by up to two orders of magnitude compared with a catalyst-free control. On the basis of these findings, we propose that reactive forms of Fe and Mn might catalyse geopolymerization in continental margin sediments and could promote OC preservation on a globally important scale.

**Fig. 1:**
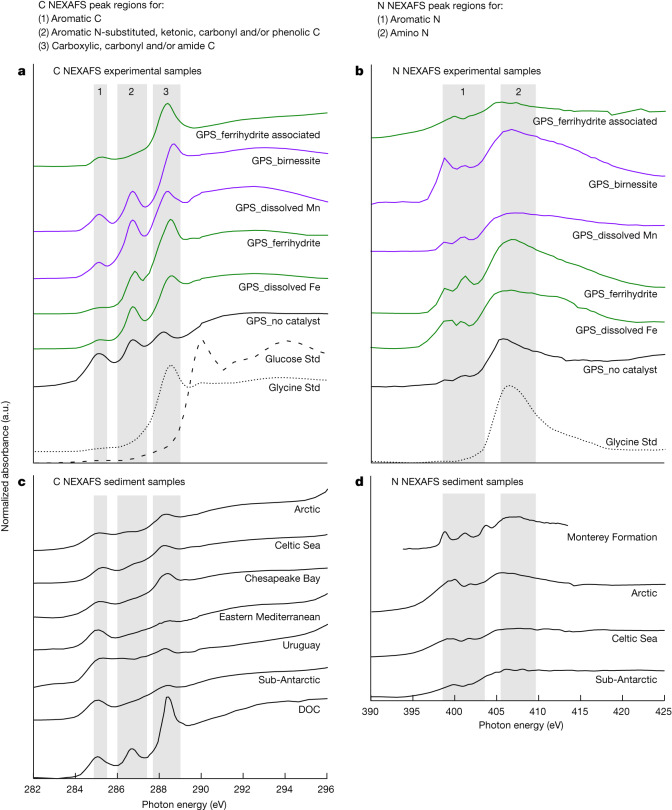
Fingerprints of C and N matched between experiments and continental margins. C and N 1s NEXAFS data plotted as energy (eV) versus normalized absorbance (presented in arbitrary units (a.u.)). Grey bands show energy regions in which spectral features associated with Maillard reaction products are expected to appear. The presence of peaks or other spectral content in these regions in both our experimental and sediment samples indicates that geopolymerization by means of a Maillard-type reaction is a likely formation pathway for persistent organics in marine sediments. **a**,**b**, C spectra (**a**) and N spectra (**b**) for glucose standard (Std), glycine standard and experimentally produced GPS in the absence (GPS_no catalyst) and presence of dissolved Fe (GPS_dissolved Fe), Fe mineral (GPS_ferrihydrite), dissolved Mn (GPS_dissolved Mn) and Mn mineral (GPS_birnessite), and associated with ferrihydrite (GPS_ferrihydrite associated). **c**,**d**, C spectra (**c**) and N spectra (**d**) for dissolved organic carbon (DOC) and continental margin sediment samples. Spectra are stacked with an arbitrary offset for clarity. Source Data

To compare the products of our experiments to dissolved OC and total OC in continental margin sediments, we used near-edge X-ray absorption fine structure (NEXAFS) spectroscopy. Results are shown in Fig. 1, whereby NEXAFS was used as a fingerprinting technique in which comparable features (for example, peaks or shoulders) in different energy regions were ascribed to a particular chemical form of OC (for example, aromatics) to indicate matching chemical structures. We fingerprinted the GPS (greater than or equal to 1,000 g mol^−1^) present in our experimental solutions separated from their respective catalysts, as well as the GPS associated with the ferrihydrite catalyst (the concentration of GPS associated with birnessite was below detection limits (Supplementary Table 1)). We then compared our GPS fingerprints to dissolved OC and total OC in our continental margin sediment samples (Supplementary Table 2). We also compared our GPS to total N in the sediment samples with sufficient N for NEXAFS measurement. Peaks and other spectral content in C regions 1, 2 and 3 (Fig. 1a) are typically assigned to aromatic C (C region 1); aromatic, aromatic N-substituted, ketonic, carbonyl and/or phenolic C (C region 2); and carboxylic, carbonyl and/or amide C (C region 3) (Supplementary Table 3). Peaks and other spectral content in N regions 1 and 2 (Fig. 1b) are typically assigned to aromatic N (N region 1) and amino N (N region 2) (Supplementary Table 4). Based on previous characterization of Maillard reaction products^16^, and thus expected Maillard molecular chemistry, we assigned peaks and other spectral content in C regions 1, 2 and 3 (Fig. 1a) to the presence of aromatic, aromatic N-substituted, carbonyl and carboxyl C (Supplementary Note 1). Similarly we assigned peaks in N regions 1 and 2 (Fig. 1b) to the presence of a variety of heterocyclic N-substituted aromatic rings and amino N^16^ (Supplementary Note 1).

We found that the spectral fingerprint of our GPS in solution (Fig. 1a) closely resembled the spectroscopic signature of dissolved OC, which also exhibited strong peaks in the spectral regions expected for OC transformation products formed by means of the Maillard reaction (Fig. 1c). The spectral fingerprint of our GPS associated with ferrihydrite (Fig. 1a,b) showed a marked amplitude dampening of the carbonyl C, aromatic N and amino N peak regions, and a shift of the carboxyl C and amino N peaks to lower energy, compared with GPS in solution. We attribute these spectral modifications to the adsorption of the carbonyl, carboxyl and amino functional groups to the mineral surface^21,22,23^. This spectral fingerprint for GPS associated with ferrihydrite is strikingly similar to the amplitude dampening of the carbonyl C, carboxyl C, aromatic N and amino N regions observed for the sediment samples, which also exhibited peaks or other spectral content in the spectral regions expected for OC transformation products formed by means of the Maillard reaction (Fig. 1c,d). Although geopolymerization is unlikely to constitute the only formation pathway for the dissolved OC and sedimentary OC pools^7^ (Supplementary Note 1 and Supplementary Fig. 2), the spectroscopic similarity between our GPS, dissolved OC and both total OC and N in continental margin sediments indicates that geopolymerization by means of a Maillard-type reaction is one viable formation pathway for refractory dissolved OC molecules^24^ and complex humic-like substances in marine sediments^5,25,26^.

**Fig. 2:**
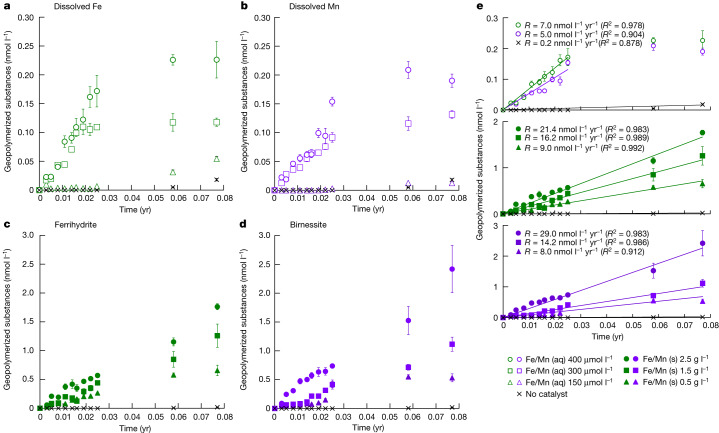
Experimental catalysis by Fe and Mn. **a**–**d**, GPS production data for the reaction of glucose and glycine under continental margin sediment temperature, plotted as time (years) versus GPS (nmol l^−1^) in the presence of dissolved Fe (**a**), dissolved Mn (**b**), Fe mineral (ferrihydrite) (**c**) and Mn mineral (birnessite) (**d**). All centre points represent the average, and error bars represent one standard error of the mean (*n* = 3). **e**, Linear regressions of data used for calculation of GPS production rate (*R*) with coefficient of determination (*R*^2^). Green and purple colour codes depict Fe and Mn data, respectively. Source Data

To measure the catalytic effect of Fe and Mn on the Maillard reaction, we used nanoparticle tracking analysis to precisely quantify the concentration of products (greater than or equal to 1,000 g mol^−1^). Under catalyst-free conditions, minimal polymerization occurred (0.2 ± 0.02 nmol l^−1^ yr^−1^ GPS). For the catalysed reaction, under anoxic conditions (Fig. 2a,b), dissolved Fe and Mn produced increased polymerization with increased catalyst concentration, generating up to an order of magnitude more GPS than the catalyst-free control (7 ± 1.05 nmol l^−1^ yr^−1^ Fe-catalysed GPS and 5 ± 0.42 nmol l^−1^ yr^−1^ Mn-catalysed GPS using 400 µmol l^−1^ catalyst). Under oxic conditions (Fig. 2c,d), ferrihydrite and birnessite produced substantially increased polymerization at all catalyst concentrations tested, which was up to an order of magnitude greater than that of dissolved Fe and Mn and two orders of magnitude greater than that of the catalyst-free control (21.4 ± 0.6 nmol l^−1^ yr^−1^ ferrihydrite-catalysed GPS and 29.0 ± 5.1 nmol l^−1^ yr^−1^ birnessite-catalysed GPS using 2.5 g l^−1^ catalyst).

**Fig. 3:**
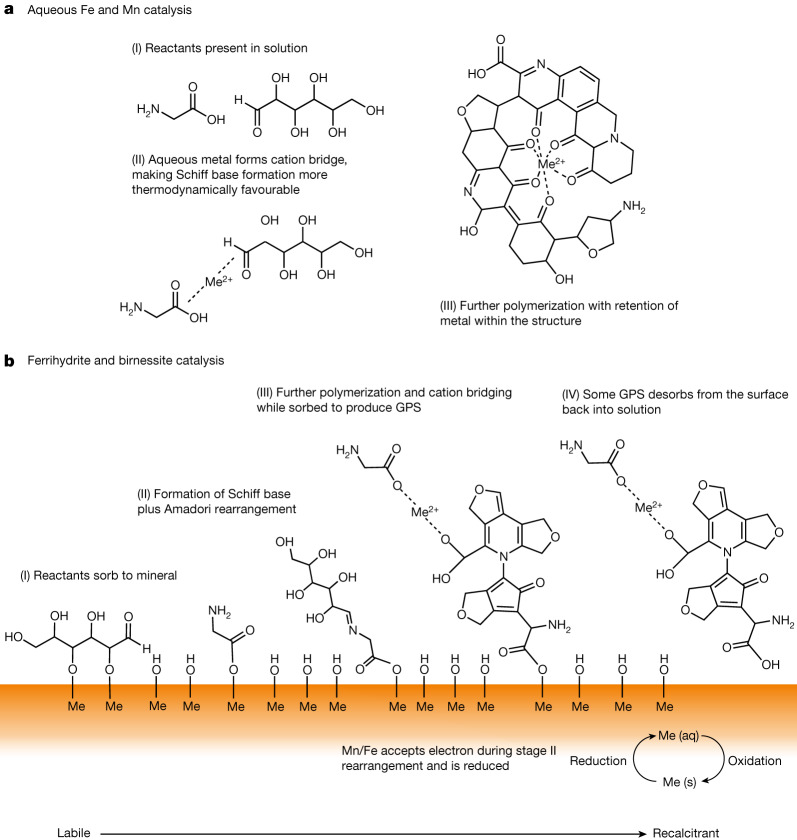
Fe and Mn catalysis of organic carbon geopolymerization. **a**, Catalysis of the Maillard reaction in association with dissolved Fe and Mn ions in solution. **b**, Catalysis of the Maillard reaction in association with reactive Fe and Mn (oxyhydr)oxide minerals.

We attribute the catalytic effect of dissolved Fe and Mn to a complexation mechanism akin to cation bridging^27^ in which these polyvalent Fe and Mn cations form stable complexes with the reactants (Fig. 3a). The bridging effect creates a more favourable free-energy reaction for Schiff base formation (the precursor to Maillard reaction products)^28^. We attribute the catalytic effect of Fe and Mn (oxyhydr)oxides to an adsorptive effect that favourably clusters and orients the reactants at the mineral surfaces, which enhances the reaction rate^21^, combined with a redox reaction between glucose and the minerals that generates dissolved Fe(II) and Mn(II) for the bridging effect^24^ (Fig. 3b). The oxidized glucose also reacts with glycine to form a Schiff base^11^.

In continental margin sediments, we posit that adsorption plays a fundamental role in the competition between geopolymerization and remineralization of reducing sugars and amino acids because adsorption can very rapidly remove reactant molecules from the microbially accessible dissolved pool and retard their remineralization^21^. In this way the adsorptive catalysis and adsorptive protection of reactants might offer a mechanism by which geopolymerization effectively competes with remineralization^22^. Following geopolymerization, dissolved Fe(II) and Mn(II) may be reoxidized and precipitate fresh mineral surfaces for further catalytic reaction. Meanwhile, negatively charged GPS^10^ could also remain adsorbed to positively charged sites at the ferrihydrite surface. These sites are abundant at our experimental and pore water pH 8 (ref. ^29^) and may offer GPS extra protection against remineralization^30^. Furthermore, GPS may desorb from the negatively charged sites at the birnessite surface, which are also abundant at pH 8 (ref. ^31^), in favour of more positively charged ferrihydrite surfaces, which could explain why we observe negligible GPS associated with birnessite (Supplementary Table 1). A positive feedback may also exist between the adsorptive catalysis of GPS and its adsorptive protection, because GPS molecules have an increased number of adsorption binding sites (Fig. 3b), and thus increased binding strength and protection from remineralization^7,22,23,32^. The production of GPS is consistent with continental margin sediment C to Fe molar ratios (of the dithionite-extractable Fe fraction) that far exceed those expected for adsorption of simple OC molecules by reactive Fe minerals (that is, greater than 1), and suggests that Fe–OC couplings might exist as macromolecular structures ‘glued’ together by Fe ions or nanoparticulate Fe (oxyhydr)oxides^19,33^.

Since we have discovered that Fe and Mn (oxyhydr)oxides catalyse the geopolymerization of OC at reaction temperatures applicable to continental margin sediments, we have used a series of evidence-based constraints in a first attempt to estimate the potential scale and importance of GPS production in oxygenated surface sediments on the continental margins (Supplementary Note 2). Using a Monte Carlo approach, we modelled spatial variation of our experimentally determined GPS production rates within a total reactive pore-water volume (1.2 × 10^14^ l), calculated using the areal extent of continental shelf (water depth of 0–200 m)^34^ and upper slope (water depth of 200–1,000 m)^35^ sediments (where more than 90% of OC is buried^4,36^) and the oxygen penetration depth (OPD), determined from an empirical relationship to water depth^37^ (Methods). We estimate that Fe and Mn mineral catalysed geopolymerization in continental margin sediments might generate and thus preserve 4.05 ± 0.55 Tg C yr^−1^ (95% confidence level). That such an amount of OC preservation might be controlled by Fe and Mn availability could have important consequences for understanding the global carbon cycle, because these elements are not usually considered by long-term carbon burial flux estimates.

In the modern Earth system, the global relationships between sedimentary OC and the individual controls of its preservation are weak, partly because there are few depositional settings that provide natural experimental analogues to disentangle the multiple factors controlling OC preservation^2^. A global relationship between sedimentary OC and Fe or Mn is therefore equally difficult to discern^19^. Over Earth’s history, preservation and burial of OC is one of the most important processes governing Earth’s long-term surface chemistry, and is the predominant source of atmospheric O_2_ over geological timescales^38^. Therefore any further control on OC preservation may have a substantial impact on how we view the evolution of Earth’s atmosphere. Although the fraction of OC burial that is attributable to GPS production might be a minor proportion of the global flux, it is still likely to be important as OC burial over long timescales is generally believed to have varied by small amounts (recording variations of only about 63 Tg C yr^−1^ over the past 300 million years^6^). By contrast, the supply of Fe and Mn catalysts has probably varied substantially over geological time, given that rates of continental erosion and of tectonic or hydrothermal input have all varied by as much as a factor of 5 over the Phanerozoic eon^39,40^, and we have shown that, with only modest changes in Fe and Mn concentrations, GPS production can increase by up to two orders of magnitude (Fig. 2).

To test the potential for sedimentary GPS formation to drive changes in Earth’s surface chemistry, we ran the SCION Earth Evolution Model^41^ over the Phanerozoic eon (Supplementary Note 3). In these model runs, we attributed 4.05 Tg C yr^−1^ of the global OC burial flux to GPS formation, and varied this GPS flux independently by a factor of 5 on the basis of maximum likely changes to global erosion rates^39^ and hydrothermal fluxes^40^. We did not make any other model alterations, but when taking the nominal 4.05 Tg C yr^−1^ GPS fraction, changes in OC burial attributed to GPS formation were able to drive changes in atmospheric O_2_ of up to 8% atm, more than one third of the present atmospheric inventory (Supplementary Fig. 3). Changes in OC preservation were also able to shift global average surface temperature by approximately 5 °C, as a result of changes to CO_2_ drawdown. Thus the GPS contribution to OC preservation and burial could be an important component of Earth’s long-term climate evolution, and one that has not yet been considered in any theoretical or numerical models of Earth’s history. Moreover, a GPS flux resulting from the catalytic effect of dissolved Fe on geopolymerization could be especially important under the ferruginous (anoxic, Fe-rich) conditions that dominated during the Precambrian Era^42^, and may have helped maintain moderate OC preservation rates and intermediate levels of atmospheric O_2_ despite low primary productivity^43^.

To conclude, we have determined a catalytic effect of Fe and Mn on OC geopolymerization at reaction temperatures (10 °C) applicable to continental margin sediments. In sediments, we posit that a combined adsorptive catalysis and adsorptive protection of reducing sugar and amino acid reactants might effectively compete with the microbial remineralization of these geopolymer building blocks^22^ such that the formation of geopolymers in sediments occurs and is much faster than previously known^7,9,18^. Our work indicates that catalysis by Fe and Mn may play a fundamental role in OC preservation, to a degree that could substantially affect the Earth’s global carbon and oxygen cycles.

## Methods

### Mineral synthesis

Ferrihydrite (2-line) was synthesized by the method of Schwertmann and Cornell^44^. Briefly, KOH solution was titrated with Fe(NO_3_)_3_·9H_2_O solution until the pH reached 7.0 ± 0.3 while stirring vigorously. The resulting precipitate was left to settle for 1–2 h before the overlying supernatant was syphoned off. The precipitate slurry was then transferred to a beaker, immersed in 5 l of ultrapure water (18.2 MΩ cm^−1^) and left to settle. The overlying supernatant was then removed and the beaker refilled with ultrapure water (18.2 MΩ cm^−^^1^). The wash cycle was repeated two or three times a day until the pH of the supernatant was between 5 and 7 (normally 3 to 4 days). The precipitate was then centrifuged at 2,000*g* for 20 min and the supernatant discarded. Disordered birnessite (δMnO_2_) was synthesized through the method of Villalobos^45^. Briefly, KMnO_4_ solution was slowly (maximum time 5 min) added to NaOH solution while stirring vigorously, then MnCl_2_.4H_2_O solution was added (maximum time 30 min) while stirring vigorously to form a black precipitate. The precipitate was left to settle for 4 h and the overlying supernatant syphoned off. The remaining precipitate was then centrifuged at 2,000*g* for 20 min and the supernatant discarded. The residue was then shaken with 1 mol l^−1^ NaCl solution for 1 h and centrifuged. The NaCl wash was repeated five times, with the final wash shaken overnight. The centrifuge–wash cycle was then repeated 10 more times with ultrapure water (18.2 MΩ cm^−^^1^) in place of NaCl, until the supernatant had a pH of approximately 12.8. The precipitate was then dialysed in ultrapure water (18.2 MΩ cm^−1^) using 12,000–14,000 g mol^−1^ of cellulose membrane tubing until the external water conductivity was less than 0.1 µS cm^−1^. Minerals were stored as wet slurries at 4 °C and mineral identity and purity were confirmed by X-ray diffraction using a Bruker D8 Diffractometer with Cu-Kα radiation (*λ* ≈ 0.154 nm). Diffractograms were recorded from 2° to 90° 2*θ* with 0.02° 2*θ* step size and 930 ms of acquisition time. Silicon dioxide was used as an analytical standard. The densities (g ml^−1^) of the final mineral precipitate slurries were determined by pipetting 1 ml of each slurry 10 times into preweighed weighing boats that were then left at 45 °C for 24 h before reweighing.

### Abiotic Fe and Mn catalyst batch experiments

Glucose and glycine were used as representative moieties for dissolved monomeric reducing sugars and dissolved free amino acids in continental margin sediment pore waters, respectively. Equimolar (0.05 M) solutions of d-glucose (Sigma Aldrich, more than 99%) and glycine (Sigma Aldrich, more than or equal to 98.5%) were made up in autoclaved Schott bottles using 10% stock solutions. For the experiments using dissolved Fe or Mn as catalysts, 2,000-ppm stock solutions of either MnCl_2_ or FeCl_2_ were added to the Schott bottles to produce concentrations of 150, 300 and 400 μM l^−1^ of Fe or Mn. For the experiments using mineral Fe or Mn as catalysts, the minerals were added to the Schott bottles to produce solid solution ratios of 0.5, 1.5 and 2.5 g l^−1^. After the experimental solutions were prepared, the pH of each experiment was measured and adjusted to pH 8.2 ± 0.1 using either NaOH or HCl buffer solutions. Experiments were then placed on a shaker table in an incubator at 10 °C and 10-ml aliquots were taken daily. Aliquot samples were centrifuged at 2,000*g* for 30 min. The centrifuged supernatants from the dissolved Fe and Mn catalyst experiments and the mineral Fe and Mn catalyst experiments were dialysed against ultrapure water (18.2 MΩ cm^−1^) using 1,000-g-mol^−1^ dialysis tubing. Dialysis was continued until the resistance of the dialyte was around 18 MΩ cm^−1^. This size dialysis tubing was chosen as an operationally defined cutoff for geopolymerized molecules produced by means of the Maillard reaction^16^. The glucose and glycine reactants are around 180 g mol^−1^ and around 75 g mol^−1^ respectively, and any unreacted glucose and glycine remaining in the experimental solutions were therefore effectively separated from the reaction products. The dialyte was kept in solution to measure the concentration of GPS and aliquots were also freeze-dried for elemental and spectroscopic analysis, as described below. The centrifuged residue from the mineral Fe and Mn catalyst experiments was also repeatedly washed in ultrapure water (18.2 MΩ cm^−1^), recentrifuged and freeze-dried for elemental and spectroscopic analysis, as described below. To ensure that the experiments proceeded abiotically, all glassware was acid-washed and autoclaved, and all stock solutions, buffer solutions and experimental solutions were prepared using autoclaved ultrapure water (18.2 MΩ cm^−1^).

### Concentration of GPS

Previous methods used to measure the production of Maillard reaction products (for example, Browning Index or E_4_/E_6_) are unable to provide absolute quantification of the geopolymers produced and also neglect GPS that are non-chromophoric^46,47^. Previous measurements of Maillard reaction rate are thus only inferred, and subsequent evaluations of the potential of the Maillard reaction to generate complex OC molecules may be underestimated^5^. To overcome these problems, we used nanoparticle tracking analysis to precisely quantify the concentration of products in the greater than or equal to 1,000-g-mol^−1^ molecular weight range. Nanoparticle tracking analysis is shown to successfully measure the concentration of Maillard reaction products^48^.

The concentration of GPS particles in the dialyte from the dissolved and mineral Fe and Mn catalyst experiments was calculated by tracking particles in a known volume of solution. Samples were diluted as required to 10^7^–10^9^ particles ml^−1^ before being immediately introduced into the sample chamber of a Malvern Nanosight NS300 (Malvern Instruments Limited) with a beam wavelength of 405 nm. Samples were then left to equilibrate for 30 s before analysis began. Each experiment was measured in triplicate with each video lasting for 215 s. To ensure that the analyses counted only organic reaction products, and not any nanoparticles of Fe or Mn mineral catalysts that may have remained in the experimental supernatants after centrifugation and subsequently passed through the 1,000-g-mol^−1^ dialysis membrane, particles that created flare and/or noise during analysis were automatically discounted by the analytical software during particle counting. Nanoparticles of Fe(III) (oxyhydr)oxides and Mn oxide have much higher refractive indices (RIs) (RI 2.32 and 3.35, respectively)^49,50^ than Maillard reaction products produced from glucose and glycine (melanoidins, RI 1.62)^51,52^, and thus create greater flare/noise during analysis. All dilutions were conducted using 0.2 μm of filtered ultrapure water (18.2 MΩ cm^−1^), which had previously been examined on the instrument to determine that it was free from contaminant nanoparticles.

### Sediment sample preparation

Bulk surface sediment samples were collected from a variety of continental margins using either multicore or grab samplers. Sediments were freeze-dried, stored at −18 °C and subsequently fumigated to remove inorganic C before NEXAFS analysis. Fumigation was achieved by weighing 20 mg of sediment into Ag cups held in a glass tray, which was then placed in a glass desiccator above a glass beaker containing 25 ml of 37% concentrated HCl for 6 h. Fumigation is shown to reduce the risk of alteration of organic molecules in coastal sediments during inorganic C removal, compared with suspension in HCl^53^.

### Carbon and nitrogen content of GPS

The C and N content of the dialyte from the dissolved and mineral Fe and Mn catalyst experiments, and the residues from the mineral Fe and Mn catalyst experiments, was determined on freeze-dried samples using a Vario PYRO cube CNS elemental analyser (Elementar).

### Molecular weight of GPS

The hydrodynamic radius of GPS was measured using dynamic light scattering (Zetasizer Nano-ZS, Model ZEN3600, Malvern Instrument Ltd). Samples were dissolved in ultrapure water (18.2 MΩ cm^−1^) and then pipetted into disposable low-volume cuvettes (ID of 1.5 cm) and measured for 180 s while keeping the solution at a constant temperature of 25 °C. The range of particle radii was found to be 3.25–4.36 nm with a maximum peak intensity at 3.77 nm. The hydrodynamic radius was then used to calculate the diffusion coefficient of GPS (*D*_GPS_) on the basis of the Stokes–Einstein equation, which in turn was used to calculate the molecular weight (MW_GPS_) following Alperin et al.^9^ (Supplementary Table 5):

$${{\rm{M}}{\rm{W}}}_{{\rm{G}}{\rm{P}}{\rm{S}}}=\frac{{R}^{3}\times {T}^{3}\times {\rho }_{{\rm{G}}{\rm{P}}{\rm{S}}}}{162{\pi }^{2}\times {N}^{2}\times {\eta }^{3}\times {{D}_{{\rm{G}}{\rm{P}}{\rm{S}}}}^{3}}$$

where *R* = gas constant; *T* = absolute temperature (K); *ρ*_GPS_ = density of GPS, which is assumed to be the same as for typical biomolecules^9,54^ (1.5 g cm^−3^); *N* = Avogadro’s constant and *η* = the dynamic viscosity of the medium.

### NEXAFS spectroscopy

The C and N 1s NEXAFS spectra of the freeze-dried dialyte from the dissolved and mineral Fe and Mn catalyst experiments, and of the freeze-dried residue from the mineral Fe catalyst experiment, were recorded on I08 beamline at Diamond Light Source Synchrotron, UK. For analysis, around 2 mg of freeze-dried sample residue was redissolved (dialyte) or suspended (residue) in 500 μl of ultrapure water (18.2 MΩ cm^−1^) water. Aliquots of 0.2 µl were then pipetted onto silicon windows (50 nm thick) and left to air-dry. Windows were glow discharged before loading with sample to improve particle distribution. Windows were then inserted into a high vacuum environment (less than 1 *×* 10^−5^ mBar) and samples were analysed in scanning transmission mode. Stacked datasets for C were collected between 275 eV and 320 eV, using varied energy resolution across 275–280 eV (1 eV), 280–284 eV (0.5 eV), 284–286.8 eV (0.2 eV), 286.8–290 eV (0.1 eV) and 290–320 eV (0.5 eV). Stacked datasets for N were collected between 385 eV and 430 eV, at a coarser energy resolution of 385–400 eV (1 eV), 400–415 eV (0.2 eV), 415–420 (0.5 eV) and 420–430 eV (1 eV) as the N 1s edge is more sensitive to beam damage. To maximize spectral resolution, the beamline uses Fresnel zone plates to focus the beam and a collimated plane grating monochromator of SX700-type with an undulator that provides a source size of 300 μm in the horizontal and 50 μm in the vertical plane, which are then refocused into a secondary source with a 50-μm slit, providing an energy resolution of better than 50 meV at the C k-edge. To minimize beam damage on the sample, dwell times were set to 10 ms per energy step following beam damage tests conducted by repeatedly measuring the same area of sacrificial samples. Beam damage manifests as a C NEXAFS peak at an absolute energy of 285.2 eV, attributable to the formation of aromatic C in the beam^55^. Sacrificial spectra with beam damage were discarded, but the position of the aromatic C peak was used for absolute energy calibration by shifting all spectra in the energy space by the required energy to align the beam damage peak to 285.2 eV. Reference spectra for the unreacted glucose and glycine were obtained from unmodified solids. The dark signal was measured routinely before the collection of sample spectra. X-ray absorption stacks were aligned using the Axis2000 software. Spectra were extracted and the dark signal was subtracted from the raw data using the Mantis software. Spectra were then exported for baseline correction, alignment, calibration and normalization using the Athena software. Baseline correction and normalization avoid spectral dependence on the total C and N content; as a result, spectral features and peak shifts are indicative of C and N molecular structure and chemistry, and not C or N concentration effects occurring during NEXAFS measurement. Peak identification for the normalized spectra was achieved with reference to literature assignations (Supplementary Tables 3 and 4).

### Application of experimental reaction rates to continental margin sediments

To provide a first attempt to estimate the potential scale and importance of GPS production in oxygenated surface sediments on the continental margins, a total reactive pore-water volume within which GPS production might occur was calculated, and the spatial variation of the experimentally determined GPS production rates within this volume were modelled as a function of pore-water and sediment properties.

To calculate the total reactive pore-water volume, the following equation was used:

$${\rm{P}}{\rm{V}}=\varphi \times {Z}_{{\rm{O}}2}\times S$$

where PV is the pore-water volume (m^3^), $\varphi$ is the porosity (dimensionless), *Z*_O2_ is the OPD (m), and *S* is the surface area of sediment (m^2^). Porosity ($\varphi$) was accounted for using a globally gridded map^14^. The impact of compaction (variability of porosity with sediment depth) in the calculation was not considered because the depth for calculation of the pore volume, that is, OPD (maximum 1.10 cm; see below), was smaller than the depth over which the porosity map has been estimated (5 cm)^56^. The model results were therefore insensitive to variations in compaction length scale, which is thought to be important over sediment depth of tens to hundreds of metres, rather than millimetres and centimetres^57^. OPD (*Z*_O2_) was determined from an empirical relationship related to water depth^37^, which was then converted to a globally gridded dataset^58^. This yielded OPDs within the range of 0.56–1.10 cm, with an average of 0.66 cm and standard deviation of 0.13 cm. Area of the sediment surface was determined from the areal extent of continental shelf (water depth of 0–200 m)^34^ and upper slope (water depth of 200–1,000 m)^35^ sediments. Using the equation and considerations above, pore-water volume was calculated in the oxic zone of each global grid point.

To model spatial variation of the experimentally determined GPS production rates as a function of continental margin sediment temperature, the Arrhenius equation and globally gridded data for continental margin bottom water temperature^59^ was used to determine GPS production rates at each grid point^60^:

$$R={R}_{{\rm{L}}{\rm{a}}{\rm{b}}}\times \,\exp \left(-\frac{{E}_{{\rm{a}}}}{{R}_{{\rm{G}}}}(\frac{1}{T}-\frac{1}{{T}_{{\rm{L}}{\rm{a}}{\rm{b}}}})\right)$$

where *R* is the GPS production rate at any given grid point (mol m^−3^ yr^−1^) and *R*_Lab_ is the GPS production rate obtained from our experiments (mol m^−3^ yr^−1^), *E*_a_ is the activation energy (J mol^−1^), *R*_G_ is the universal gas constant (J K^−1^ mol^−1^), *T* is the absolute temperature (K) at any given grid point and *T*_Lab_ is the absolute temperature (K) of our experiments (283.15 K). The temperature map was obtained from the interpolation of data for global bottom water temperature, which is commonly used as the temperature of surface sediments^59^. The GPS production rates were determined from the linear regressions of the experimental reaction rates using each of the mineral catalyst concentrations (that is, 0.5, 1.5 and 2.5 g l^−1^) for both mineral Fe and mineral Mn (Fig. 2). Only the mineral Fe- and Mn-catalysed GPS production rates, which might effectively compete with microbial remineralization^22^, were used in the determination. The activation energy was taken from a collated list of Maillard reaction studies^61^. Regarding other spatially variable parameters, sedimentation rates strongly correlate with water column depth^37^, whereas bio-irrigation/bioturbation depths/coefficients are mostly synchronized and correlate with OPD^62,63^. The relationship between water column depth and OPD is used explicitly in the calculation of the total reactive pore-water volume as described above, and thus variations in sedimentation rate and bio-irrigation/bioturbation are implicitly included in our approach.

To estimate how much carbon might be preserved in sediments on the continental shelf and upper slope (water depth of 0–1,000 m) as a result of GPS formation, the above equation was integrated into the following:

$${C}_{{\rm{p}}{\rm{r}}{\rm{e}}{\rm{s}}}={{\rm{M}}{\rm{W}}\times {C}_{{\rm{c}}{\rm{o}}{\rm{n}}{\rm{t}}}\times {\rm{P}}{\rm{V}}\times R}_{{\rm{L}}{\rm{a}}{\rm{b}}}\times \,\exp \left(-\frac{{E}_{{\rm{a}}}}{{R}_{{\rm{G}}}}(\frac{1}{T}-\frac{1}{{T}_{{\rm{L}}{\rm{a}}{\rm{b}}}})\right)$$

where *C*_pres_ is the rate of carbon preservation (g C yr^−1^) as a result of GPS production, MW is the molecular weight of GPS (g mol^−1^) and *C*_cont_ is the carbon content of GPS (wt%). The molecular weight of GPS was determined from the experimental particle hydrodynamic radii measured using dynamic light scattering, which was then used to calculate the diffusion coefficient of GPS on the basis of the Stokes–Einstein equation, which in turn was used to calculate the molecular weight^9^ (Methods and Supplementary Table 5). The C content of GPS was determined from the experimental elemental analysis (Methods and Supplementary Table 1).

The rate of C preservation was determined in a Monte Carlo procedure in which the input dataset described above was run 1,000 times for each grid point of the global map (which is more than one million nodes for 0.25° × 0.25° resolution). In this approach, the input parameters for GPS production rates, GPS activation energy, GPS molecular weight and GPS C content were varied over a range determined either during the experiments or in the literature. Specifically, the range for GPS production rates was varied between those determined for the lowest and highest mineral catalyst concentrations (0.5–2.5 g l^−1^); for GPS activation energy was varied between a collated list of Maillard reaction activation energies, determined for a range of amino acid and reducing sugar pairings^61^; for GPS molecular weight was varied over the range of hydrodynamic radii measured using dynamic light scattering; and for GPS C content was varied over the instrument uncertainty (Supplementary Table 6). In the Monte Carlo procedure, the maximum and minimum of each experimental or literature range was then further increased or decreased by one standard deviation, respectively, in an attempt to reasonably cover the broadest extent of input parameter possibilities that may be encountered in margin sediments. If the latter led to a value less than zero, a value close to zero (10^−15^) was selected instead (Supplementary Table 6). The final data were generated randomly on the basis of a uniform distribution within the selected ranges for each parameter. At each grid point, after 1,000 Monte Carlo runs, the mean rate of C preservation (g C yr^−1^) as a result of GPS production was returned by the model. The uncertainties were determined from the confidence intervals on the basis of a 95% confidence level according to the 2.5th and 97.5th percentiles of the Student’s *t*-distribution. The sum of C preserved as a result of GPS production at all of the grid points active in the analysis yielded the global annual rate of C preserved as a result of GPS production.

## Online content

Any methods, additional references, Nature Portfolio reporting summaries, source data, extended data, supplementary information, acknowledgements, peer review information; details of author contributions and competing interests; and statements of data and code availability are available at 10.1038/s41586-023-06325-9.

## Supplementary information

Supplementary Information This file contains Supplementary Figs. 1–3, Tables 1–6 and Notes 1–3.

Supplementary Data Fig. 2 Underlying source data for Supplementary Fig. 2.

## Data Availability

All nanoparticle tracking analysis data and NEXAFS data are available at figshare (10.6084/m9.figshare.22496791). The underlying data are also associated with the online version of this article as part of the Supplementary Information. Source data are provided with this paper.

## Source data

Source Data Fig. 1
Source Data Fig. 2

## Code availability

The pore-water model scale-up code is publicly available on figshare (10.6084/m9.figshare.22491844). The SCION code used to test the potential for sedimentary GPS formation to drive changes in Earth’s surface chemistry over the Phanerozoic is also publicly available on figshare (10.6084/m9.figshare.22496176).
