# Supplementary material for: Long-term organic carbon preservation enhanced by iron and manganese
Source: Nature. 2023 Aug 2;621(7978):312–7. doi: 10.1038/s41586-023-06325-9 (PMC10499600; doi:10.1038/s41586-023-06325-9)
Supplement: Supplementary file 1 — This file contains Supplementary Figs. 1–3, Tables 1–6 and Notes 1–3. [file 41586_2023_6325_MOESM1_ESM.docx]

*Supplementary Information*

**Long-term organic carbon preservation enhanced by iron and manganese**

Oliver W. Moore^a*^, Lisa Curti^a^, Clare Woulds^b^, James A. Bradley^c,d^, Peyman Babakhani^a^, Benjamin J.W. Mills^a^, William B. Homoky^a^, Ke-Qing Xiao^a,e^, Andrew W. Bray^a^, Ben J. Fisher^a,f^, Majid Kazemian^g^, Burkhard Kaulich^g^, Andrew W. Dale^h^, Caroline L. Peacock^a^

^a^ School of Earth and Environment, University of Leeds, Leeds, LS2 9JT, UK

^b^ School of Geography, University of Leeds, Leeds, LS2 9JT, UK

^c^ School of Geography, Queen Mary University of London, Mile End Road, London, E1 4NS, UK

^d^ Department of Geochemistry, GFZ, German Research Centre for Geosciences, Potsdam, Germany

^e^ Research Center for Eco-Environmental Sciences, Chinese Academy of Sciences, Beijing, China

^f^ School of GeoSciences, University of Edinburgh, Edinburgh, UK

^g^ Diamond Light Source Ltd., Harwell Science and Innovation Campus, Didcot, OX11 0DE, UK

^h^ GEOMAR Helmholtz Centre for Ocean Research Kiel, Kiel, Germany

*corresponding author

**Supplementary Note 1: Extended discussion of the NEXAFS data:** Determining a precise chemical signature of GPS and OC is hampered by the fact that these compounds are resistant to hydrolysis, and thus difficult to analyse via traditional techniques^1, 2^, resulting in >70% of OC in sediments being classed as the molecularly uncharacterised component (MUC) ^3^. Many analytical techniques also generate artifacts of the same chemical signatures that might be expected for complex organics^1^. In this study we use the non-destructive method of near-edge X-ray absorption fine structure (NEXAFS) spectroscopy to compare the chemical signature of our GPS to both total OC and N in margin sediments from a global sample set (Fig. 1 main text; Supplementary Table 2). We chemically characterise the GPS present in the experimental solutions (separated from their respective catalyst) and also any OC and N associated with the solid residue in the ferrihydrite catalysed experiments. We compare these to sediment OC and N from continental shelf settings (water depth <200 m) across a range of latitudes from 76^o^ N (Arctic) to 54^o^ S (Sub-Antarctic) and a range of ages from modern (Sub-Antarctic, Uruguay, Eastern Mediterranean, Chesapeake Bay, Celtic Sea and Arctic) to Miocene sediments (Monterey Formation deposited between 6-17 Ma) which represent a spectrum of organic sources and ages (Fig. 1 main text; Supplementary Table 2).

The C 1s NEXAFS spectra of our GPS present in the experimental solutions (Fig. 1a main text) show three broad peaks in regions corresponding to (Supplementary Table 3):

*aromatic C* (C region 1: comprising C 1s π* transitions associated with C=C at 284.9 – 285.5 eV);

*aromatic, aromatic N-substituted, ketonic, carbonyl and/or phenolic C* (C region 2: comprising C 1s π* transitions associated with aromatic and aromatic N-substituted C=C, C=O, C-N, C=N at 286 – 286.5 eV, and with aromatic, ketonic, carbonyl and phenolic C=O, R-(C=O)-R’, C=C-OH at 286.5 – 287.4 eV);

*carboxyl, carbonyl and/or amide C* (C region 3: comprising C 1s π* transitions associated with R-COOH, COO, C=O, NH_2_-C=O, R-(NH_2_)-R’ at 287.7 – 289.0 eV).

The N 1s NEXAFS spectra of our GPS (Fig. 1b main text) show two broad peaks in regions corresponding to (Supplementary Table 4):

*aromatic N* (N region 1: comprising N 1s π* transitions associated with pyridinic, pyrazinic and pyramidinic N=C at 398.6 – 402.2 eV, and with pyrrolic, pyrazolic and imadazolic N-C at 400 – 403.5 eV);

*amino N* (N region 2: comprising N 1s σ* transitions associated with N-C, N-H at 405.5 – 409.7 eV) .

End products of the Maillard reaction are very complex and not yet completely characterised^4^, but are reported to contain aromatic C compounds in the form of furans (heterocyclic 5-membered aromatic rings, containing 4 C and 1 O) consistent with C region 1, carbonyl compounds (C=O) consistent with C region 2, and a variety of heterocyclic N-substituted aromatic rings, including pyridines (heterocyclic 6-membered aromatic rings, containing 5 C and 1 N), pyrazines (heterocyclic 6-membered aromatic rings, containing 4 C and 2 N) and pyrroles (heterocyclic 5-membered aromatic rings, containing 4 C and 1 N) all consistent with the presence of one broad or multiple more defined peaks in N region 1^4-7^.

We therefore assign peaks or significant spectral content in C region 1 and 2 to aromatic C and carbonyl compounds, respectively. Similarly we assign peaks or significant spectral content in N region 1 to heterocyclic N compounds. The C peaks in C region 3 (carboxylic, carbonyl and/or amide C) and the N peaks in N region 2 (amino N) likely reflect structural features (i.e., carboxyl C and amino N) retained in the GPS from the glycine reactant. Crucially, the C peaks in regions 1 and 2 and the N peaks in region 1, reflect increased complexity and do not appear in the glucose and glycine reactants.

The C spectral fingerprint of our GPS in solution (Fig. 2a main text) closely resembles the C spectroscopic signature of dissolved OC, which also exhibits strong peaks in the C spectral regions expected for OC transformation products formed via the Maillard reaction (Fig. 1c main text). We were unable to collect high quality N NEXAFS for the dissolved organic sample.

The C and N spectral fingerprints for our GPS associated with ferrihydrite (Fig. 1a, b main text) closely resemble the C and N spectroscopic signatures of continental margin sediment, which also exhibit peaks or significant spectral content in the spectral regions expected for OC transformation products formed via the Maillard reaction (Fig. 1c, d main text). The C spectral fingerprint for GPS associated with ferrihydrite shows a marked amplitude dampening of the carbonyl peak (C region 2) and a shift of the carboxyl peak (C region 3) to lower energy, compared to our GPS in solution (Fig. 1a main text). Deconvolution of the carbonyl and carboxyl peak is complicated by the dominance of the latter, and it is therefore inconclusive as to whether the carbonyl peak has also been broadened and/or shifted into the carboxyl peak. Either way, a dampening, broadening and/or shifting of the carbonyl and carboxyl peaks indicate that the C=O and COOH functional groups are interacting with the mineral surface^8^. Complex organics (ciprofloxacin and norfloxacin) are reported to sorb to Fe (oxyhydr)oxides through ligand exchange between the oxygens of the carbonyl and carboxyl groups and the oxygens of mineral –FeOH sorption sites^9,10^. The carboxyl-mineral bonds may also result in a broadening of the carbonyl peak as a result of the electron-withdrawing nature of the carboxyl-iron bonds, as is shown to occur with other similarly complex organic molecules (gatifloxacin and ciprofloxacin) ^11,12^. Alternatively, electrostatic interactions such as hydrogen bonding or ion-dipole interactions are shown to occur between the ketone group and the hydroxylated surfaces of clay minerals^13,14^. Interactions such as these would weaken the C=O bond, which is shown to shift the carbonyl peak in Fourier transform infrared (FTIR) spectroscopy^14^.

The N spectral fingerprint for our GPS associated with ferrihydrite also shows a marked amplitude dampening of the aromatic peak region (N region 1) and both a dampening and a shift of the amino peak (N region 2) to lower energy, compared to our GPS in solution (Fig. 1b main text). Amino acids are known to interact with metal oxide surfaces via strong electrostatic interactions^15-18^ and similar changes and shifts in the amino peak are observed in FTIR spectra of amino acids sorbed to mineral oxides^19,20^. The concentrations of C and N associated with birnessite are too low to collect NEXAFS (Supplementary Table 1).

The spectroscopic similarity we show between our GPS, dissolved OC and both total OC and N in continental margin sediments (Fig. 1 main text), indicates that geopolymerisation via a Maillard-type reaction is one viable formation pathway for refractory dissolved OC molecules^21^ and complex humic-like substances in marine sediments^22-24^; the latter of which show a very similar C NEXAFS spectroscopic chemical fingerprint to our GPS and continental margin sediment samples, most notably with the presence of significant spectral content in C region 2^24^. In marine sediments however, geopolymerisation is unlikely to constitute the only formation pathway for refractory dissolved OC and complex sedimentary OC, and several others possible formation pathways are suggested^2^. Perhaps most obviously, peaks or significant spectral content in the spectral regions expected for Maillard reaction products (C region 2 and N region 1) might also result from the presence of primary biomolecules and their constituent parts. It is worth noting however, that the spectral signatures of dissolved OC and both sediment OC and N are difficult to construct using a combination of only these molecules. Significant spectral content in the carbonyl C region (C region 2) is inconsistent with the primary biomolecules (proteins, polysaccharides and lipids) ^24^, and content in both C region 2 and N region 1 is also inconsistent with the most prevalent amino acids in marine sediments (aspartic acid, glycine, alanine) ^25^ (Supplementary Fig. 2c). Some aromatic amino acids (most notably histidine) show peaks in C region 2 (Supplementary Fig. 2c) and N region 1 (Supplementary Fig. 2d), the latter attributable to pyramidinic N^26^, but these do not comprise a significant fraction of the sediment amino acid profile^25^. Spectral content in C region 2 and N region 1 may also result from the presence of nucleic acids or nucleic acid fragments, whose nucleobase building blocks contain carbonyl C at ~286.6 eV that can be shifted up to ~289 eV when N is added to the carbonyl group^27,28^, and both pyramidinic and imadazolic N that also manifest as peaks in the pyridinic, pyrazinic and pyrrolic N region^29,30^ (Supplementary Fig. 2d). Nucleic acids and nucleobases are extremely labile however, and their persistence as a significant component of sedimentary organics is unlikely^31^. Instead GPS and other geopolymerised complex organics might form from the constituent parts of nucleic acids during abiotic conversion reactions that occur during their decomposition^23^.

**Supplementary Note 2: Extended discussion of the application of experimental reaction rates to continental margin sediments:** It is important to note that our estimate for Fe and Mn mineral-catalysed geopolymerisation in continental margin sediments carries a number of limitations and uncertainties inherent in the application of our experimentally determined GPS production rates to natural sediments. Here we provide a thorough discussion of the primary limitations and uncertainties associated with our scale-up and suggest how further work might address these.

To initiate the Maillard reaction in our experiments we use the monomeric reducing sugar glucose and the free amino acid glycine as reactants. We use glucose and glycine as representative moieties for all monomeric reducing sugars and all free amino acids, respectively, because all reducing sugars and free amino acids can take part in the Maillard reaction^4,32^, and we use these representatives at millimolar concentration (50 mmol L^-1^). Glucose and glycine are commonly present in margin porewaters (glucose is normally the major monomeric sugar^33^ and glycine is one of the most common free amino acids within marine sediments^3^) but their specific reactivity towards the Maillard reaction may not be representative of other reactant pairings within the monomeric reducing sugar and free amino acid reactant classes, and in margin porewaters glucose and glycine and their respective reactant classes are present at micromolar to sub-micromolar concentration^34-36^. Because reaction rates can be a function of reactant properties and concentrations, the application of our experimental GPS production rates, determined using glucose and glycine at elevated concentration, to margin sediments containing a mixture of monomeric reducing sugars and free amino acids at reduced concentration, therefore contains an inherent uncertainty.

Determining Maillard reaction kinetics is difficult because the reaction is extremely complex^4^. For many reactant pairings, formation of the more stable intermediate and advanced Maillard products are reported to obey zero-order kinetics with respect to the reactants, and colour formation (which is the most studied aspect of the Maillard reaction) is also typically modelled as a zero-order reaction^37^. The occurrence of a zero-order reaction is due to the fact that the concentration of reactants present is usually quite high compared to the concentration of intermediates and advanced products formed, hence the concentration of reactants is essentially constant relative to the products^37^. In our experiments the concentration of glucose and glycine (50 mmol L^-1^) is very high compared to the concentration of GPS formed (a few nmol L^-1^ GPS, Figure 2 main text) and suggests that our glucose and glycine reactant pairing also obeys zero-order kinetics, in which the reaction rate is independent of the concentration of reactants, providing a critical threshold concentration of reactants is met.

The critical threshold concentration of glucose and glycine required to sustain our sum experimental GPS production rate is ~2 micromolar of both glucose and glycine (calculated using the average molecular weight of our GPS (27067 g mol^-1^; Supplementary Table 5) and considering that glucose (180 g mol^-1^) and glycine (75 g mol^-1^) are combined in a ~1:1 ratio in a GPS unit (Supplementary Table 1) such that a GPS unit equates to ~255 g mol^-1^, (although Fe mineral catalysts have also been shown to catalyse glucose-glucose polymerisation^38^), and every 1 mole of GPS contains ~106 GPS units and thus ~53 moles of both glucose and glycine; therefore our average GPS production rates via Fe and Mn mineral catalysis (15.5 x 10^-9^ mol L^-1^ yr^-1^ for Fe-catalysed and 17.1 x 10^-9^ mol L^-1^ yr^-1^ for Mn-catalysed presented in Supplementary Table 6) require ~8 x 10^-7^ mol L^-1^ yr^-1^ of both glucose and glycine (for Fe-catalysed) and ~9 x 10^-7^ mol L^-1^ yr^-1^ of both glucose and glycine (for Mn-catalysed)). Approximately 2 micromolar probably exceeds the concentration of glucose and almost certainly exceeds the concentration of glycine in typical margin porewaters (field measurements of individual monomeric reducing sugars and free amino acids are extremely scarce but for the available data glycine concentrations are micromolar to sub-micromolar^35^). It is thus unlikely that our experimental GPS production rates are applicable to margin sediments considering the Maillard reaction of only glucose and glycine. Approximately ~2 micromolar however, is within the range of concentrations reported for the monomeric reducing sugar and free amino acid reactant classes in margin porewaters (field measurements for these reactant classes are particularly scarce in oxic margin sediments within which we postulate our GPS production to occur, but glucose can comprise the majority of dissolved free carbohydrates in oxic margin porewater up to ~6 micromolar^34^ while free amino acids at oxic sites can be up to ~10 micromolar^35^). We therefore posit that our experimental GPS production rates are applicable to margin sediments considering the Maillard reaction of monomeric reducing sugars and free amino acids, and to try and account for natural variability in the specific reactivity of different reactant pairings within these reactant classes, we include a range of Maillard reaction activation energies for a range of different reactant pairings (including reducing sugars, amino acids and proteins) ^37^, which we increase by plus/minus one standard deviation in our Monte Carlo approach to further account for natural variability (see Methods).

To catalyse the Maillard reaction in our experiments we use synthetic ferrihydrite and birnessite. Whilst ferrihydrite and birnessite are present in oxygenated surficial margin sediments^15^, our synthetic versions may not be representative of the concentrations and reactivities of ferrihydrite and birnessite present in these settings. Because our experimental GPS production rates are a function of catalyst concentration, their application to margin sediments therefore contains an inherent uncertainty. Determining the concentration of ferrihydrite in sediments is difficult because the Fe (oxyhydr)oxide fraction is most usually targeted using the dithionite extraction, which isolates poorly crystalline phases like ferrihydrite as well as more crystalline Fe oxides, which might be less catalytically reactive^39^. To our knowledge there are no down core global data sets for Fe extracted using a weaker treatment, such as Fe extracted by hydroxylamine-HCL, which is likely more suitable for constraining the concentration of ferrihydrite. Determining the concentration of birnessite in sediments is similarly difficult. In our experiments to try and approximate the concentration of ferrihydrite within margin sediments (i.e., within the OPD used for our scale-up – maximum 1.10 cm, average 0.66 cm) we consider that, hypothetically, if all dithionite extractable Fe in margin sediments (0.73 wt% Fe^40^) was ferrihydrite, this would equate to ~6.9 g L^-1^ ferrihydrite within these settings (calculated using a typical porosity of margin sediments of ~0.8 and thus a sediment-porewater ratio of ~0.2:0.8^41,42^, equating to 250 cm^3^ of sediment per 1 L of porewater; a typical density of dry shelf sediment of ~2.7 gdw cm^-3^ and thus ~675 gdw of sediment per 1 L of porewater; a dithionite-extractable Fe concentration of 0.73 wt% for margin sediments^40^ and thus a hypothetical (assuming all Fe is ferrihydrite) solid-solution ratio of ferrihydrite in typical margin sediments of ~6.9 g L^-1^ (based on a generic formula of Fe_2_O_3_⋅0.5H_2_O)). Then given that only some fraction of dithionite extractable Fe is ferrihydrite within margin sediments, we use our ferrihydrite catalyst over a concentration range (0.5 – 2.5 g L^-1^) that approximates the low end of dithionite extractable Fe within these settings. To approximate the concentration of birnessite within margin sediments we consider that, if the majority of ascorbic acid extractable Mn in surface oxic margin sediments (0.2 – 0.5 wt% Mn) ^43^ is birnessite (where birnessite has the generic formula: Na_0.7_Ca_0.3_Mn_7_O_14_·2.8H_2_O), this would equate to ~2.1 – 5.9 g L^-1^ birnessite within these settings. Then to facilitate comparison with the ferrihydrite experiments we also use our birnessite catalyst over a concentration range (0.5 – 2.5 g L^-1^) that approximates the low end of Mn concentrations within margin sediments.

Further variability in ferrihydrite and birnessite concentrations and reactivities exist within oxygenated surficial margin sediments, for example, due to variations in oxygen concentrations within the oxic layer and the transformation of these poorly crystalline phases to more crystalline oxides with time before their burial below the oxic layer. The latter scenario is potentially mitigated to some extent because the sedimentary redox cycling of Fe during early diagenesis is estimated to occur hundreds of times prior to burial below the redoxcline and might therefore maintain mineral reactivity^44^. In our scale-up to try and account for natural variability in the concentration and reactivity of our ferrihydrite and birnessite catalysts, we increase the range of our experimental GPS production rates, initially determined over our experimental catalyst concentration ranges, by plus/minus one standard deviation in our Monte Carlo approach (Methods main text).

Given our discussion of the primary limitations and uncertainties associated with our scale-up above, we present our scale-up as a first attempt to estimate the potential scale and significance of GPS production in oxygenated surface sediments on the continental margins. Each of the input variables to our scale-up are evidence-based but subject to debate, and their globally applicable ranges will ultimately only be resolved with further experimentation and/or field measurements. We suggest than in the first instance experimental efforts should focus on using reducing sugar and amino acid reactant moieties (including polymeric forms) at different concentrations and with different reactivity towards the Maillard reaction, and using other Fe oxide catalysts at different concentrations and with different crystallinity to constrain their catalytic activity. Field measurements could try to extend the available data for monomeric reducing sugar and free amino acid concentrations in margin porewaters, and more routinely use a weaker extraction treatment to more suitably target ferrihydrite in margin sediments. These data should help refine future estimates of GPS production in margin sediments and might decrease or increase the scale and significance of GPS production in these settings and thus the contribution of Fe and Mn minerals to the global carbon cycle.

**Supplementary Note 3: Effects of changes to the long-term organic carbon burial rate on Earth’s surface environment:** In this study we estimate that 4.05 **±** 0.55 Tg C Yr^-1^ of global OC burial may be attributed to GPS formation, and thus may be controlled by mechanisms which are different to those commonly assumed for OC preservation. To explore what this might mean for the evolution of Earth’s surface conditions over geological time, we run the SCION (Spatial Continuous Integration) Earth Evolution Model^45^. SCION reconstructs long-term climate and biogeochemistry, in order to explore the processes which have regulated Earth’s surface conditions over the Phanerozoic Eon (the last ~540 million years). It is an extension of the COPSE^46^ and GEOCARB^47^ models which uses a climate component based on GEOCLIM^48^. For full description of the model and comparisons to the geological record see Mills et al^45^.

Here we make a simple modification to SCION by splitting the OC preservation (= burial) flux into two fluxes. We assume that some fraction of OC preservation is driven by GPS formation, while the remainder follows the original model and is related to marine new production and sediment bioturbation. The total OC burial rate at the present day is therefore unchanged. To explore what effects the changes in GPS preservation over geological time might have, we run the model under a constant 5-fold increase or decrease in GPS preservation. This represents the likely maximum changes to the delivery of Fe to the oceans over Phanerozoic time, based on changes at subduction, ridge and rift boundaries (approx. 0.5 - 5 x present day^49^; or global erosion rates (approx. 0.2 – 1 x present day^50^).

The model results are shown in Supplementary Fig. 3. When OC preservation is increased, atmospheric O_2_ concentration is substantially higher in the model. This is because greater rates of OC burial result in greater net accumulation of oxygen. Carbon dioxide is also generally higher in this scenario because a higher O_2_ abundance results in a greater rate of continental OC oxidation, and limits the terrestrial biosphere through photorespiration and wildfire activity. Decreases in OC preservation have the opposite effect, but are less powerful because total OC preservation cannot be reduced by more than the entire GPS fraction. These changes can be more powerful between about 400 Ma and 150 Ma, where the largely arid supercontinent Pangaea weakened the silicate weathering feedback and resulted in poorer climate regulation. Before Devonian time the changes to the CO_2_ greenhouse and temperature are minimised due to the lack of a productive terrestrial biosphere, but O_2_ remains highly variable.

Our analysis here demonstrates that changing OC preservation independently of other processes can cause major changes in a long-term model of the global carbon cycle, even when other stabilizing feedbacks are considered. Moreover considering previously unrecognised variations in GPS preservation means that the mismatches between the model and the proxy record can potentially be better reconciled. For example, the modified model better matches the higher temperatures and oxygen levels suggested by proxies for the Mesozoic, indicating that high rates of hydrothermal Fe input (due to higher seafloor production^51^) may have played a role in raising organic carbon burial rates, oxygen concentrations and surface temperatures during this time. This preliminary analysis indicates that the greatest GPS-driven change to the Earth system may be achieved when hydrothermal or continental Fe fluxes are greatly elevated. This is especially intriguing during the Precambrian where hydrothermal fluxes may have been 10 or even 100 times greater than the present day^52-54^, and thus much greater Fe-promoted preservation of OC may have helped maintain relatively high atmospheric O_2_ levels despite the less productive^55,56^ primitive biosphere.

**Supplementary Table 1.** Bulk elemental analyses. Analyses of C and N for the experimental solutions (dialyte) from the dissolved Fe and Mn catalyst batch experiments, and the experimental solutions (dialyte) and solid residue (after centrifugation) from the Fe and Mn mineral catalyst batch experiments, after 28 days reaction time at 10^o^C. Uncertainty is shown as one standard error of the mean (1SEM), with all measurements performed in triplicate (n = 3); B.D. = below detection.

The C/N ratio of our GPS (mean C/N 9.7) shows it is consistent with dissolved porewater organic matter (C/N ~7 – 20) ^3^ and bulk organic matter (C/N ~10 – 13) ^57^ in margin sediments and open ocean surface marine sediments (C/N ~8 – 12) ^3^. The mean GPS C/N suggests that glucose (C= 6; N= 0) and glycine (C= 2; N= 1) are combined into GPS in a ~1:1 ratio. It should be noted that the C/N ratio of the residues reflects both GPS, and also any unreacted glucose and/or glycine that are associated with the mineral particles.

|  | **Catalyst** | **Catalyst Concentration** |  | **C (%)** | **1SEM** | **N (%)** | **1SEM** | **C/N** |
| --- | --- | --- | --- | --- | --- | --- | --- | --- |
|  | **None** | N/A | Dialyte | 33.49 | 2.88 | 2.18 | 0.26 | 15.4 |
| **Iron** | **Mineral (g/L)** | 0.5 | Residue | 4.36 | 0.03 | 0.62 | 0.01 | 7.0 |
|  |  |  | Dialyte | 33.12 | 0.43 | 2.11 | 0.31 | 15.7 |
|  |  | 1.5 | Residue | 4.01 | 0.06 | 0.58 | 0.01 | 6.9 |
|  |  |  | Dialyte | 31.48 | 0.24 | 2.33 | 0.10 | 13.5 |
|  |  | 2.5 | Residue | 0.40 | 0.00 | 0.02 | 0.00 | 20.2 |
|  |  |  | Dialyte | 35.76 | 0.08 | 2.45 | 0.15 | 14.6 |
|  | **Dissolved (μM)** | 150 | Dialyte | 34.82 | 0.25 | 5.89 | 0.43 | 5.9 |
|  |  | 300 | Dialyte | 34.67 | 0.14 | 6.10 | 0.32 | 5.7 |
|  |  | 400 | Dialyte | 35.40 | 0.03 | 5.58 | 0.01 | 6.3 |
| **Manganese** | **Mineral (g/L)** | 0.5 | Residue | B.D. | - | B.D. | - | - |
|  |  |  | Dialyte | 33.48 | 0.10 | 5.64 | 0.03 | 5.9 |
|  |  | 1.5 | Residue | B.D. | - | B.D. | - | - |
|  |  |  | Dialyte | 33.87 | 0.07 | 5.48 | 0.31 | 6.2 |
|  |  | 2.5 | Residue | B.D. | - | B.D. | - | - |
|  |  |  | Dialyte | 36.23 | 0.07 | 2.96 | 0.02 | 12.2 |
|  | **Dissolved (μM)** | 150 | Dialyte | 33.21 | 0.23 | 5.23 | 0.33 | 6.3 |
|  |  | 300 | Dialyte | 31.46 | 0.09 | 4.88 | 0.33 | 6.4 |
|  |  | 400 | Dialyte | 32.16 | 4.70 | 4.83 | 0.36 | 6.7 |

**Supplementary Table 2**. Continental shelf sediments investigated with NEXAFS spectroscopy.

We compare the spectral fingerprint of our GPS to the spectroscopic signature of total OC and N in continental margin sediments from a global sample set, as well as to marine DOC. Our samples are all from continental shelf settings (water depth ≤200 m) across a range of latitudes from 76^o^ N (Arctic) to 54^o^ S (Sub-Antarctic) and a range of ages from recent sediments (Antarctic, Uruguay, Eastern Seaboard, Celtic Sea and Arctic, ~0 kyr), Holocene sediments (Eastern Mediterranean, ~3.8 kyr) to Miocene sediments^23,58^ (Monterey Formation deposited between 6-17 Ma).

| **Location** | **Latitude (°N)** | **Longitude (°E)** | **TOC (dwt%)** |
| --- | --- | --- | --- |
| Sub-Antarctic | -54.16 | -37.98 | 1.22 |
| Uruguay | -36.46 | -53.16 | 1.09 |
| Eastern Mediterranean | 32.01 | 34.16 | 0.56 |
| Chesapeake Bay (NIST1941b) | 39.20 | -76.52 | 2.99 |
| Celtic Sea | 51.20 | -6.13 | 1.34 |
| Arctic | 76.49 | 30.50 | 2.60 |
| Miocene Monterey Formation | 36.36 | -121.53 | 6.69 |
| HMW Marine DOC | 8.28 to 10.00 | -90.00 to -87.00 | ~35.00 |

**Supplementary Table 3.** C 1s NEXAFS approximate energy ranges and assignments of primary absorption peaks.

| **Peak Energy (eV)** | **Transition** | **Bond** | **C Forms** |
| --- | --- | --- | --- |
| 283.0 – 284.5 | 1s-π* | C=O | Aromatic / Quinonic |
| 284.9 – 285.5 | 1s-π* | C=C | Aromatic |
| 285.8 – 286.2 | 1s-π* | C=O | Aromatic |
| 286.0 – 286.5 | 1s-π* | C=C  C=O  C-N  C=N | Aromatic / Aromatic substituted with N |
| 286.5 – 287.4 | 1s-π* | C=O  R-(C=O)-R’  C=C-OH | Aromatic / Ketonic / Carbonyl / Phenolic |
| 287.6 – 288.2 | 1s-3p/σ* | C-H | Aliphatic |
| 287.7 – 289.0 | 1s-π* | R-COOH  COO  C=O  NH_2_-C=O  R-(NH_2_)-R’ | Carboxylic / Carbonyl / Amide |
| 289.2 – 290.2 | 1s-π*  1s-3p/σ* | C-OH  C=O  R-O-R’ | Polysaccharide / Alcohol / Carbonyl / Hydroxylated and Ether-linked |

Sources: Peak assignments are based on data collected from the literature^59-80^. Note inorganic carbonate exhibits a distinctive π* resonance at 290.3 – 290.6 eV.

**Supplementary Table 4.** N 1s NEXAFS approximate energy ranges and assignments of primary absorption peaks.

| **Peak Energy (eV)** | **Transition** | **Bond** | **N Forms** |
| --- | --- | --- | --- |
| 398.6 – 402.2 | 1s-π* | N=C | Aromatic (Pyridinic, Pyrazinic, Pyramidinic) |
| 400.0 – 403.5 | 1s-π* | N-C | Aromatic (Pyrrolic, Pyrazolic, Imadazolic) |
| 400.7 – 402.5 | 1s-π* | NH_2_-C=O | Amide |
| 405.5 – 409.7 | 1s-σ* | N-C  N-H | Amino |

Sources: Peak assignments are based on data collected from the literature^26,29,30,81,82^ and references therein.

**Supplementary Table 5.** Molecular weight of GPS determined from the particle hydrodynamic radii measured by Dynamic Light Scattering. The range of particle radii was found to be 3.25 – 4.36 nm with a maximum peak intensity at 3.77 nm. The hydrodynamic radius was then used to calculate the diffusion coefficient of GPS based on the Stokes-Einstein equation, which in turn was used to calculate the molecular weight^83^ (see Methods in main text).

|  | **Hydrodynamic Radius (nm)** | **Diffusion Coefficient (cm^2^ yr^-1^)** | **Molecular weight (g mol^-1^)** |
| --- | --- | --- | --- |
| Nominal | 3.77 | 19.96 | 27067 |
| Maximum | 4.36 | 17.26 | 41867 |
| Minimum | 3.25 | 23.16 | 17341 |

**Supplementary Table 6. Experimentally or literature determined range and extended range for the variable input parameters used in the Monte Carlo analysis.** ^*^Indicates cases where one standard deviation less than the original range minimum fell lower than zero and thus a low value of 10^-15^ was chosen.

|  |  |  |  | **Monte Carlo range** | | |  |
| --- | --- | --- | --- | --- | --- | --- | --- |
| **Parameter** | **Symbol** | **Unit** | **Experimentally or literature determined range** | **mean** | **min** | **max** | ***Note** |
| Experimental GPS production rate with Fe catalysis | *R_Lab_* | nmol L^-1^ yr^-1^ | 9, 16.2, 21.4 | 15.5 | 2.77 | 27.6 | Experimentally determined (Figure 3) |
| Experimental GPS production rate with Mn catalysis | *R_Lab_* | nmol L^-1^ yr^-1^ | 8, 14.2, 29 | 17.1 | 1.0E-15^*^ | 39.8 | Experimentally determined (Figure 3 ) |
| Arrhenius activation energy | *Ea* | kJ mol^-1^ | 23–238 | 131 | 1.0E-15^*^ | 390 | From literature^60^ |
| Molecular weight | *MW* | g mol^-1^ | 17341– 41867 | 3.0E+4 | 1.0E-15^*^ | 5.9E+4 | Experimentally determined (Supplementary Table 5). |
| Carbon content when using Fe catalysis | *C_Cont_* | wt% | 35.68– 35.84 | 35.8 | 35.6 | 36.0 | Experimentally determined (Supplementary Table 1) |
| Carbon content when using Mn catalysis | *C_Cont_* | wt% | 36.16– 36.30 | 36.2 | 36.2 | 36.3 | Experimentally determined (Supplementary Table 1) |

**Supplementary Figure 1:** Schematic showing simplified Maillard Reaction, in which the carbonyl group of a sugar reacts with the amino group of an amino acid, to form water and an unstable Schiff base of glycosylamine; the glycosylamine then undergoes Amadori rearrangement to form an amino ketone compound; the Amadori rearrangement product then undergoes a range of other rearrangement and condensation reactions to form a plethora of different products that contain aromatic C and N-substituted rings with carbonyl, carboxyl and amino functional groups^4^.

**
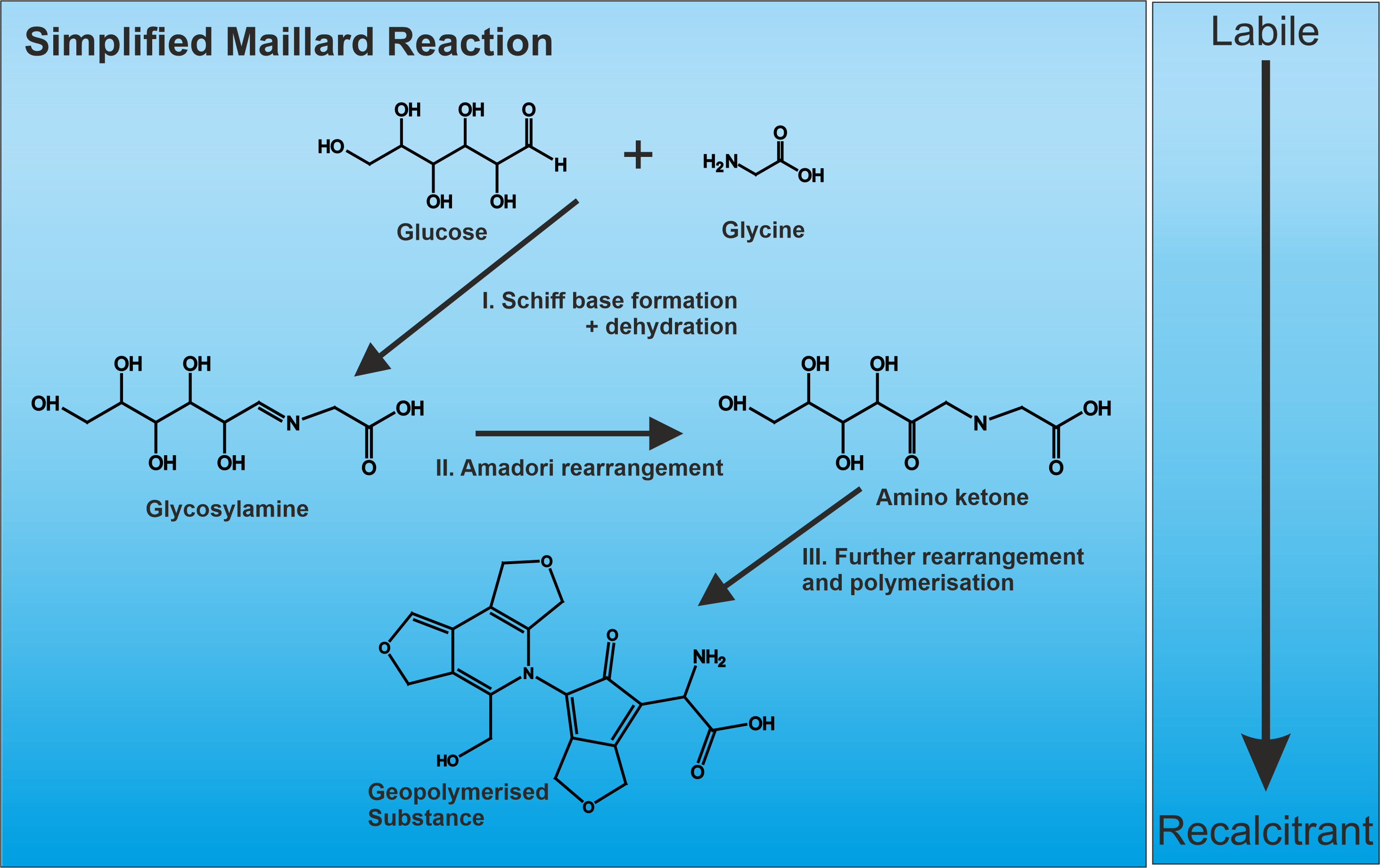
**

**Supplementary Figure 2:** C and N 1s NEXAFS data plotted as energy (eV) vs. normalised absorbance (arbitrary units). Grey bands show energy regions in which spectral features associated with Maillard reaction products are expected to appear. **a.** C spectra and **b.** N spectra for glucose, glycine and experimentally produced geopolymerised carbon (GPS) in the absence (GPS_no catalyst) and presence of dissolved Fe (GPS_dissolved Fe), Fe mineral (GPS_ferrihydrite), dissolved Mn (GPS_dissolved Mn), Mn mineral (GPS_birnessite), and associated with ferrihydrite (GPS_ferrihydrite associated); **c.** C spectra and **d.** N spectra for biopolymers, amino acids, nucleotide bases and nucleic acids. Panel **a.** also shows C spectra for experimentally produced GPS using glucose and either lysine (GPS_no catalyst_lys) or aspartic acid (GPS_no catalyst_asp) with the same spectral features as experimentally produced GPS using glucose and glycine (GPS_no catalyst).

Biopolymer spectra for protein (bovine albumin serum), polysaccharide (alginate) and lipid (1,2-dipalmitoyl-sn-glycero-3-phosphoethanolamine) are reproduced here from the literature (C spectra^84^ and protein N spectrum^85^), where all spectra were recorded in transmission mode. Amino acid spectra for alanine and aspartic acid, and the aromatic amino acids histidine, phenylalanine, tyrosine and tryptophan are reproduced here from the literature^26^, where spectra were recorded in partial electron yield (PEY) mode. Nucleotide base spectra for cytosine, uracil, thymine, guanine and adenine are reproduced here from the literature^29^, where spectra were recorded in PEY mode. Nucleic acids DNA and RNA are reproduced here from the literature and were recorded in transmission mode (DNA C spectrum) ^84^ or total fluorescence yield (TFY) mode (DNA and RNA N spectra) ^86^.

It should be noted that significant spectral content in the carbonyl C region (C region 2) is inconsistent with the primary biomolecules (proteins, polysaccharides and lipids) ^24^, and content in both C region 2 and N region 1 is also inconsistent with the most prevalent amino acids in marine sediments (aspartic acid, glycine, alanine) ^25^. It should also be noted that while some aromatic amino acids possess a C NEXAFS peak or some spectral content in C region 2 (histidine, tyrosine and tryptophan) and N NEXAFS peaks in N region 1 (histidine and tryptophan), these amino acids are generally present as only very minor components of the marine sediment amino acid profile^25^. Finally while spectral content in C region 2 and N region 1 may also result from the presence of nucleic acids or nucleic acid fragments, these are extremely labile, and their persistence as a significant component of sedimentary organics is unlikely^31^.

We compare only spectral peak positions between the literature spectra and our experimentally produced geopolymerised carbon (GPS) spectra, although literature spectra collected in transmission mode are comparable to our spectra for both spectral peak positions and amplitudes. Spectra are stacked with an arbitrary offset for clarity.

**Supplementary Figure 3.** SCION Earth Evolution Model with inclusion of changes to OC burial attributed to GPS production. Here the present day GPS burial flux is assumed to be 4.05 Tg C yr^-1^. Black line shows the original SCION baseline over Phanerozoic time^45^, pink solid line shows GPS burial fraction treated independently of other model OC burial and held at 5 $\times$ present day rate, blue line shows the same scenario with GPS burial held at $\frac{1}{5}\times$ present day rate. **A.** atmospheric O_2_, **B.** atmospheric CO_2_, and **C.** global average surface temperature. In all panels yellow shows the proxy data compilation^87-91^.


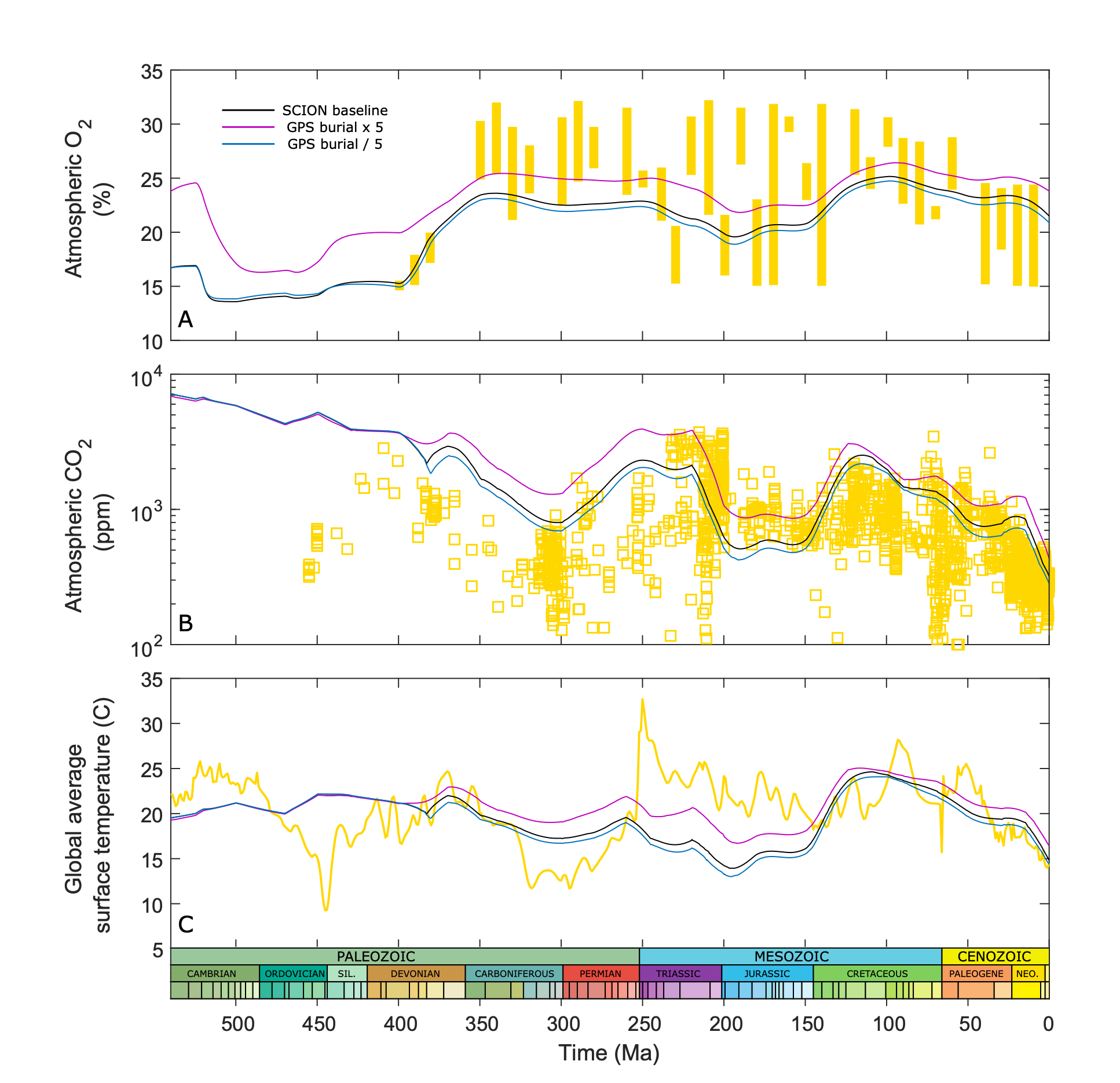


**References**

1 Hedges, J. Polymerization of humic substances in natural environments. *Humic substances and their role in the environment*, 605-625 (1988).

2 Burdige, D. Preservation of organic matter in marine sediments: controls, mechanisms, and an imbalance in sediment organic carbon budgets? *Chemical reviews* **107**, 467-485 (2007).

3 Burdige, D. *Geochemistry of marine sediments*. (Princeton University Press, 2006).

4 Wang, H.-Y., Qian, H. & Yao, W.-R. Melanoidins produced by the Maillard reaction: Structure and biological activity. *Food chemistry* **128**, 573-584 (2011).

5 Adams, A., Abbaspour Tehrani, K., Keršienė, M., Venskutonis, R. & De Kimpe, N. Characterization of model melanoidins by the thermal degradation profile. *Journal of Agricultural and Food chemistry* **51**, 4338-4343 (2003).

6 Adams, A., Borrelli, R., Fogliano, V. & De Kimpe, N. Thermal degradation studies of food melanoidins. *Journal of Agricultural and Food Chemistry* **53**, 4136-4142 (2005).

7 Ames, J., Bailey, R. & Mann, J. Analysis of Furanone, Pyranone, and New Heterocyclic Colored Compounds from Sugar− Glycine Model Maillard Systems. *Journal of agricultural and food chemistry* **47**, 438-443 (1999).

8 Chen, C., Dynes, J., Wang, J. & Sparks, D. Properties of Fe-organic matter associations via coprecipitation versus adsorption. *Environmental science & technology* **48**, 13751-13759 (2014).

9 Gu, C. & Karthikeyan, K. Sorption of the antimicrobial ciprofloxacin to aluminum and iron hydrous oxides. *Environmental science & technology* **39**, 9166-9173 (2005).

10 Al-Mustafa, J. & Tashtoush, B. Iron (II) and iron (III) perchlorate complexes of ciprofloxacin and norfloxacin. *Journal of Coordination Chemistry* **56**, 113-124 (2003).

11 Trivedi, P. & Vasudevan, D. Spectroscopic investigation of ciprofloxacin speciation at the goethite− water interface. *Environmental science & technology* **41**, 3153-3158 (2007).

12 Li, X. & Bi, E. Different surface complexation patterns of gatifloxacin at typical iron mineral/water interfaces. *Environmental Earth Sciences* **78**, 630 (2019).

13 Parfitt, R. & Mortland, M. Ketone Adsorption on Montmorillonite 1. *Soil Science Society of America Journal* **32**, 355-363 (1968).

14 Kohl, R. & Taylor, S. Hydrogen bonding between the carbonyl group and Wyoming bentonite. (1960).

15 Lambert, J.-F. Adsorption and polymerization of amino acids on mineral surfaces: a review. *Origins of Life and Evolution of Biospheres* **38**, 211-242 (2008).

16 Grafe, M., Eick, M., Grossl, P. & Saunders, A. Adsorption of arsenate and arsenite on ferrihydrite in the presence and absence of dissolved organic carbon. *Journal of environmental quality* **31**, 1115-1123 (2002).

17 Benetoli, L. *et al.* Amino acid interaction with and adsorption on clays: FT-IR and Mössbauer spectroscopy and X-ray diffractometry investigations. *Origins of Life and Evolution of Biospheres* **37**, 479-493 (2007).

18 Schwertmann, U., Wagner, F. & Knicker, H. Ferrihydrite–humic associations. *Soil Science Society of America Journal* **69**, 1009-1015 (2005).

19 Schwaminger, S. *et al.* Nature of interactions of amino acids with bare magnetite nanoparticles. *The Journal of Physical Chemistry C* **119**, 23032-23041 (2015).

20 Meng, M., Stievano, L. & Lambert, J.-F. Adsorption and thermal condensation mechanisms of amino acids on oxide supports. 1. Glycine on silica. *Langmuir* **20**, 914-923 (2004).

21 Huang, P. Abiotic catalysis. *Handbook of Soil Science* **200**, 303-334 (2000).

22 Hedges, J. The formation and clay mineral reactions of melanoidins. *Geochimica et Cosmochimica Acta* **42**, 69-76 (1978).

23 Vairavamurthy, A. & Wang, S. Organic nitrogen in geomacromolecules: insights on speciation and transformation with K-edge XANES spectroscopy. *Environmental science & technology* **36**, 3050-3056 (2002).

24 Brandes, J. *et al.* Examining marine particulate organic matter at sub-micron scales using scanning transmission X-ray microscopy and carbon X-ray absorption near edge structure spectroscopy. *Marine Chemistry* **92**, 107-121 (2004).

25 Lee, C., Wakeham, S. & Hedges, J. Composition and flux of particulate amino acids and chloropigments in equatorial Pacific seawater and sediments. *Deep Sea Research Part I: Oceanographic Research Papers* **47**, 1535-1568 (2000).

26 Zubavichus, Y., Shaporenko, A., Grunze, M. & Zharnikov, M. Innershell absorption spectroscopy of amino acids at all relevant absorption edges. *The Journal of Physical Chemistry A* **109**, 6998-7000 (2005).

27 Samuel, N., Lee, C.-Y., Gamble, L., Fischer, D. & Castner, D. NEXAFS characterization of DNA components and molecular-orientation of surface-bound DNA oligomers. *Journal of electron spectroscopy and related phenomena* **152**, 134-142 (2006).

28 Solomon, D. *et al.* Carbon (1s) NEXAFS spectroscopy of biogeochemically relevant reference organic compounds. *Soil Science Society of America Journal* **73**, 1817-1830 (2009).

29 Zubavichus, Y., Shaporenko, A., Korolkov, V., Grunze, M. & Zharnikov, M. X-ray absorption spectroscopy of the nucleotide bases at the carbon, nitrogen, and oxygen K-edges. *The Journal of Physical Chemistry B* **112**, 13711-13716 (2008).

30 Lehmann, J. *et al.* Synchrotron-based near-edge X-ray spectroscopy of natural organic matter in soils and sediments. *Biophysico-chemical processes involving natural nonliving organic matter in environmental systems*, 729-781 (2009).

31 Middelburg, J. *Marine Carbon Biogeochemistry: A Primer for Earth System Scientists*. (Springer, 2019).

32 Hodge, J. Dehydrated foods, chemistry of browning reactions in model systems. *Journal of agricultural and food chemistry* **1**, 928-943 (1953).

33 Arnosti, C. & Holmer, M. Carbohydrate dynamics and contributions to the carbon budget of an organic-rich coastal sediment. *Geochimica et Cosmochimica Acta* **63**, 393-403 (1999).

34 Jensen, M., Holmer, M. & Thamdrup, B. Composition and diagenesis of neutral carbohydrates in sediments of the Baltic-North Sea transition. *Geochimica et Cosmochimica Acta* **69**, 4085-4099 (2005).

35 Mintrop, L. & Duinker, J. Depth profiles of amino-acids in porewater of sediments from the Norwegian-Greenland Sea. *Oceanologica acta* **17**, 621-631 (1994).

36 Burdige, D. & Martens, C. Biogeochemical cycling in an organic-rich coastal marine basin: 11. The sedimentary cycling of dissolved, free amino acids. *Geochimica et Cosmochimica Acta* **54**, 3033-3052 (1990).

37 Van Boekel, M. Kinetic aspects of the Maillard reaction: a critical review. *Food/Nahrung* **45**, 150-159 (2001).

38 Singh, K., Prasad, M. & Kumar, D. Role of Iron in Catalysing the Caramelization of Sugar and its Preventative and Curative Measures. *Proc. of 8th Joint Conv. of three Associations*, 47-62 (2005).

39 Poulton, S. & Canfield, D. Development of a sequential extraction procedure for iron: implications for iron partitioning in continentally derived particulates. *Chemical geology* **214**, 209-221 (2005).

40 Anderson, T. & Raiswell, R. Sources and mechanisms for the enrichment of highly reactive iron in euxinic Black Sea sediments. *American Journal of Science* **304**, 203-233 (2004).

41 Klump, J. & Martens, C. The seasonality of nutrient regeneration in an organic‐rich coastal sediment: Kinetic modeling of changing pore‐water nutrient and sulfate distributions. *Limnology and Oceanography* **34**, 559-577 (1989).

42 Mulsow, S., Boudreau, B. & Smith, J. Bioturbation and porosity gradients. *Limnology and Oceanography* **43**, 1-9 (1998).

43 Lenstra, W., Klomp, R., Molema, F., Behrends, T. & Slomp, C. A sequential extraction procedure for particulate manganese and its application to coastal marine sediments. *Chemical Geology* **584**, 120538 (2021).

44 Homoky, W. *et al.* Iron colloids dominate sedimentary supply to the ocean interior. *Proceedings of the National Academy of Sciences* **118** (2021).

45 Mills, B., Donnadieu, Y. & Goddéris, Y. Spatial continuous integration of Phanerozoic global biogeochemistry and climate. *Gondwana Research* (2021).

46 Bergman, N., Lenton, T. & Watson, A. COPSE: a new model of biogeochemical cycling over Phanerozoic time. *American Journal of Science* **304**, 397-437 (2004).

47 Berner, R. GEOCARBSULF: a combined model for Phanerozoic atmospheric O2 and CO2. *Geochimica et Cosmochimica Acta* **70**, 5653-5664 (2006).

48 Goddéris, Y., Donnadieu, Y., Le Hir, G., Lefebvre, V. & Nardin, E. The role of palaeogeography in the Phanerozoic history of atmospheric CO2 and climate. *Earth-Science Reviews* **128**, 122-138 (2014).

49 Merdith, A., Williams, S., Brune, S., Collins, A. & Müller, R. Rift and plate boundary evolution across two supercontinent cycles. *Global and planetary change* **173**, 1-14 (2019).

50 Hay, W. *et al.* Evaporites and the salinity of the ocean during the Phanerozoic: Implications for climate, ocean circulation and life. *Palaeogeography, Palaeoclimatology, Palaeoecology* **240**, 3-46 (2006).

51 Marcilly, C. M., Torsvik, T. H. & Conrad, C. P. Global Phanerozoic sea levels from paleogeographic flooding maps. *Gondwana Research* (2022).

52 Schopf, J. & Klein, C. *The Proterozoic biosphere: a multidisciplinary study*. (Cambridge University Press, 1992).

53 Kharecha, P., Kasting, J. & Siefert, J. A coupled atmosphere–ecosystem model of the early Archean Earth. *Geobiology* **3**, 53-76 (2005).

54 Alcott, L., Mills, B. & Poulton, S. Stepwise Earth oxygenation is an inherent property of global biogeochemical cycling. *Science* **366**, 1333-1337 (2019).

55 Laakso, T. & Schrag, D. The role of authigenic carbonate in Neoproterozoic carbon isotope excursions. *Earth and Planetary Science Letters* **549**, 116534 (2020).

56 Planavsky, N. *et al.* Evolution of the structure and impact of Earth’s biosphere. *Nature Reviews Earth & Environment*, 1-17 (2021).

57 Keil, R., Tsamakis, E., Fuh, C., Giddings, J. & Hedges, J. Mineralogical and textural controls on the organic composition of coastal marine sediments: Hydrodynamic separation using SPLITT-fractionation. *Geochimica et Cosmochimica Acta* **58**, 879-893 (1994).

58 JARVIE, D. & LUNDELL, L. of Organic Matter in the Miocene Monterey Formation. *The Monterey Formation: from rocks to molecules*, 268 (2001).

59 Hitchcock, A. & Ishii, I. Carbon K-shell excitation spectra of linear and branched alkanes. *Journal of Electron Spectroscopy and Related Phenomena* **42**, 11-26 (1987).

60 Robin, M., Ishii, I., McLaren, R. & Hitchcock, A. Fluorination effects on the inner-shell spectra of unsaturated molecules. *Journal of Electron Spectroscopy and Related Phenomena* **47**, 53-92 (1988).

61 Francis, J. & Hitchcock, A. Inner-shell spectroscopy of p-benzoquinone, hydroquinone, and phenol: distinguishing quinoid and benzenoid structures. *The Journal of Physical Chemistry* **96**, 6598-6610 (1992).

62 Hitchcock, A., Urquhart, S. & Rightor, E. Inner-shell spectroscopy of benzaldehyde, terephthalaldehyde, ethylbenzoate, terephthaloyl chloride and phosgene: models for core excitation of poly (ethylene terephthalate). *The Journal of Physical Chemistry* **96**, 8736-8750 (1992).

63 Boese, J., Osanna, A., Jacobsen, C. & Kirz, J. Carbon edge XANES spectroscopy of amino acids and peptides. *Journal of Electron Spectroscopy and Related Phenomena* **85**, 9-15 (1997).

64 Cody, G., Botto, R., Ade, H. & Wirick, S. The application of soft X-ray microscopy to the in-situ analysis of sporinite in coal. *International Journal of Coal Geology* **32**, 69-86 (1996).

65 Cody, G., Ade, H., Wirick, S., Mitchell, G. & Davis, A. Determination of chemical-structural changes in vitrinite accompanying luminescence alteration using C-NEXAFS analysis. *Organic Geochemistry* **28**, 441-455 (1998).

66 Scheinost, A., Kretzschmar, R., Christl, I. & Jacobsen, C. *Carbon group chemistry of humic and fulvic acid: A comparison of C-1s NEXAFS and 13C-NMR spectroscopies*. 39-47 (Royal Society of Chemistry, 2001).

67 Stöhr, J. *et al.* Liquid crystal alignment on carbonaceous surfaces with orientational order. *Science* **292**, 2299-2302 (2001).

68 Ade, H. & Urquhart, S. NEXAFS spectroscopy and microscopy of natural and synthetic polymers. *In Chemical Applications of Synchrotron Radiation, Advanced Series in Physical Chemistry, Vol. 12, Sham, T. K., ed., World Scientific Publishing, River Edge, NJ* **285** (2002).

69 Kaznacheyev, K. *et al.* Innershell absorption spectroscopy of amino acids. *The Journal of Physical Chemistry A* **106**, 3153-3168 (2002).

70 Schäfer, T., Hertkorn, N., Artinger, R., Claret, F. & Bauer, A. in *Journal de Physique IV (Proceedings).* 409-412 (EDP sciences).

71 Schäfer, T. *et al.* Origin and mobility of fulvic acids in the Gorleben aquifer system: implications from isotopic data and carbon/sulfur XANES. *Organic Geochemistry* **36**, 567-582 (2005).

72 Cooney, R. & Urquhart, S. Chemical trends in the near-edge X-ray absorption fine structure of monosubstituted and para-bisubstituted benzenes. *The Journal of Physical Chemistry B* **108**, 18185-18191 (2004).

73 Hitchcock, A., Stöver, H., Croll, L. & Childs, R. Chemical mapping of polymer microstructure using soft X-ray spectromicroscopy. *Australian journal of chemistry* **58**, 423-432 (2005).

74 Lehmann, J. *et al.* Near‐edge X‐ray absorption fine structure (NEXAFS) spectroscopy for mapping nano‐scale distribution of organic carbon forms in soil: Application to black carbon particles. *Global Biogeochemical Cycles* **19** (2005).

75 Schumacher, M., Christl, I., Scheinost, A., Jacobsen, C. & Kretzschmar, R. Chemical heterogeneity of organic soil colloids investigated by scanning transmission X-ray microscopy and C-1s NEXAFS microspectroscopy. *Environmental science & technology* **39**, 9094-9100 (2005).

76 Solomon, D., Lehmann, J., Kinyangi, J., Liang, B. & Schäfer, T. Carbon K‐edge NEXAFS and FTIR‐ATR spectroscopic investigation of organic carbon speciation in soils. *Soil Science Society of America Journal* **69**, 107-119 (2005).

77 Solomon, D. *et al.* Molecular signature and sources of biochemical recalcitrance of organic C in Amazonian Dark Earths. *Geochimica et cosmochimica Acta* **71**, 2285-2298 (2007).

78 Heymann, K., Lehmann, J., Solomon, D., Schmidt, M. & Regier, T. C 1s K-edge near edge X-ray absorption fine structure (NEXAFS) spectroscopy for characterizing functional group chemistry of black carbon. *Organic Geochemistry* **42**, 1055-1064 (2011).

79 Prietzel, J. *et al.* Comparison of soil organic carbon speciation using C NEXAFS and CPMAS 13C NMR spectroscopy. *Science of The Total Environment* **628**, 906-918 (2018).

80 Brandes, J., Wirick, S. & Jacobsen, C. Carbon K-edge spectra of carbonate minerals. *Journal of synchrotron radiation* **17**, 676-682 (2010).

81 Leinweber, P. *et al.* in *Developments in Soil Science* Vol. 34 255-288 (Elsevier, 2010).

82 Zubavichus, Y., Shaporenko, A., Grunze, M. & Zharnikov, M. NEXAFS spectroscopy of homopolypeptides at all relevant absorption edges: Polyisoleucine, polytyrosine, and polyhistidine. *The Journal of Physical Chemistry B* **111**, 9803-9807 (2007).

83 Alperin, M., Albert, D. & Martens, C. Seasonal variations in production and consumption rates of dissolved organic carbon in an organic-rich coastal sediment. *Geochimica et Cosmochimica Acta* **58**, 4909-4930 (1994).

84 Toner, B. *et al.* Preservation of iron (II) by carbon-rich matrices in a hydrothermal plume. *Nature Geoscience* **2**, 197-201 (2009).

85 Keiluweit, M. *et al.* Nano-scale investigation of the association of microbial nitrogen residues with iron (hydr) oxides in a forest soil O-horizon. *Geochimica et Cosmochimica Acta* **95**, 213-226 (2012).

86 Leinweber, P. *et al.* Nitrogen K-edge XANES–an overview of reference compounds used to identifyunknown'organic nitrogen in environmental samples. *Journal of synchrotron radiation* **14**, 500-511 (2007).

87 Glasspool, I. & Scott, A. Phanerozoic concentrations of atmospheric oxygen reconstructed from sedimentary charcoal. *Nature Geoscience* **3**, 627-630 (2010).

88 Scotese, C., Song, H., Mills, B. & van der Meer, D. Phanerozoic paleotemperatures: The earth’s changing climate during the last 540 million years. *Earth-Science Reviews*, 103503 (2021).

89 Cather, S., Dunbar, N., McDowell, F., McIntosh, W. & Scholle, P. Climate forcing by iron fertilization from repeated ignimbrite eruptions: The icehouse–silicic large igneous province (SLIP) hypothesis. *Geosphere* **5**, 315-324 (2009).

90 Foster, G., Royer, D. & Lunt, D. Future climate forcing potentially without precedent in the last 420 million years. *Nature communications* **8**, 1-8 (2017).

91 Witkowski, C., Weijers, J., Blais, B., Schouten, S. & Damsté, J. Molecular fossils from phytoplankton reveal secular PCO2 trend over the Phanerozoic. *Science advances* **4**, eaat4556 (2018).
